# A Theoretical Study on the Antioxidant Activity of Piceatannol and Isorhapontigenin Scavenging Nitric Oxide and Nitrogen Dioxide Radicals

**DOI:** 10.1371/journal.pone.0169773

**Published:** 2017-01-09

**Authors:** Yang Lu, AiHua Wang, Peng Shi, Hui Zhang

**Affiliations:** 1 College of Material Science and Engineering, Harbin University of Science and Technology, Harbin, People’s Republic of China; 2 College of Chemical and Environmental Engineering, Harbin University of Science and Technology, Harbin, People’s Republic of China; University of Calgary, CANADA

## Abstract

The antioxidant activity of naturally occurring stilbene compounds piceatannol (PIC) and isorhapontigenin (ISO) scavenging two free radicals (NO and NO_2_) were studied using density functional theory (DFT) method. Four reaction mechanisms have been considered: hydrogen atom transfer (HAT), radical adduct formation (RAF), single electron transfer (SET), and sequential proton loss electron transfer (SPLET). The reaction channels in water solution were traced independently, and the respective thermodynamic and kinetic parameters were obtained. We found PIC and ISO scavenge NO mainly through RAF mechanism, and scavenge NO_2_ through HAT mechanism. The capacity of PIC scavenging NO_2_ is much higher than ISO, but the reactivity of scavenging NO is lower than ISO.

## Introduction

Reactive nitrogen species (RNS) are nitrogen-centered radicals and related species produced by normal cellular metabolism of human body. Nitric oxide radical (NO) is a highly RNS produced by the oxidation of the terminal guanido-nitrogen atoms of L-arginine in organism [1]. In different micro-environment, NO can be converted to various other RNS such as nitrosonium cation (NO^+^), nitroxyl anion (NO^-^) and peroxynitrite (ONOO^-^) [2]. Nitric dioxide radical (NO_2_) is formed from the reaction of peroxyl radical and NO, polluted air or smoking [3]. Overproduction of RNS is called nitrosative stress [4–5]. Nitrosative stress may initiate the nitrosylation reaction that can change the structures of proteins and so inhibit their normal function. Nitrosative stress can also cause damage to membrane fatty acids, DNA and its repair mechanism. Thus, it is significant to find natural antioxidants that can help in scavenging the excess RNS and then avoid oxidative damage in humans.

Piceatannol (4’,5’,3,5-tetrahydroxystilbene) is a natural occurring stilbene compound widespread in various plant species, such as grape [6], peanut [7], vaccinium berries [8], euphorbia lagascae [9], etc. It has been reported that PIC has numerous beneficial effects on some age-related diseases, such as anti-inflammatory, anticarcinogenic, antiviral, antioxidative, neuroprotective and estrogenic properties [10–16]. As a potential antioxidant, it has been demonstrated that PIC has the ability of scavenging diverse free radicals such as hydroxyl, peroxyl, superoxide and lipid peroxyl radical [17–19]. It has also been reported that PIC can protect against DNA damage caused by hydroxyl radicals in L1210, K562 and HL-60 leukemic cells [20]. Moreover, PIC can suppress reactive oxygen radical generation and increase the GSH/GSSG ratio in B16 melanoma cells [21].

Isorhapontigenin (3,4’,5-trihydroxy-3’-methoxy-stilbene) is also a natural stilbene compound, which can be isolated from Chinese herb Belamcanda chinensis [22] and rhubarb [23]. Wine grapes are main dietary sources of PIC, and recently ISO were also identified from wine grapes [24]. ISO shows potent antioxidant activity in vitro, with activity being higher than that shown by vitamin E [25–26]. It has been reported ISO can inhibit the respiratory burst of PMA-activated rat neutrophils through scavenging oxygen free radicals [27]. ISO could also prevent the oxidation of human LDL and other prooxidant systems in vivo [26, 28].

Except above experimental studies, some theoretical investigation on the radical scavenging activity of PIC and ISO have been completed. Rossi et al. researched the reaction mechanism of PIC and resveratrol (RES) scavenging ·OH and ·OOH by DFT method, they concluded PIC is a more efficient scavenger than RES [29]. Cordova-Gomez et al. investigated the scavenging ability of PIC and RES toward ·OOH in water and lipid solution, using DFT method and SMD solvation model. They found PIC is a better ·OOH scavenger than RES in both media [30]. Perez-Gonzalea et al. evaluated the free radical scavenging activity of a series of polyphenols, PIC is a good antioxidant among them, because having a smaller bond dissociation energy [31]. It is can be found that these research are concerned only with ROS, however RNS as another kind of harmful radical in vivo were ignored. As for as ISO, in previous our team theoretically investigated the antioxidant mechanisms of ISO and PIC scavenging two ROS (·OH and ·OOH) [32], no studies addressed the scavenging activity of ISO toward some specific radicals until quite recently.

Therefore, the aim of the present work is to carry out a theoretical study on the activity of PIC and ISO scavenging two NOS (NO and NO_2_) by the quantum mechanics-based test for overall free radical scavenging activity (QM-ORSA) protocol. QM-ORSA is a reliable approach to identify the chemical compounds with the highest activity under different conditions [33]. In this study, all possible active sites of PIC and ISO scavenging NO and NO_2_ have been examined, the thermodynamic and kinetic data of the corresponding channels in water solvent have been obtained to identify the main mechanism of the antioxidant reactions.

## Computational Methods

All electronic calculations have been performed with the package of program GAUSSIAN 09 [34], using the DFT M05-2X functional and the 6–311++G(d,p) basis set. M05-2X functional has been chosen as it yields satisfactory overall performance for the thermodynamic and kinetic calculations in organic and biological systems involving free radical reactions [35–37]. Unrestricted wave function was employed for the open-shell system. Geometry optimization and frequency calculation of all stationary points (reactants, complexes, transition states and products) were identified. Intrinsic reaction coordinate (IRC) calculations have been performed to confirm that the transition states (TS) properly connect reactants and products.

Solvent effects were introduced as single point calculations on the optimized gas-phase geometries in the framework of the continuum solvation model based on solute electron density (SMD), which is recommended by Gaussian Manual to compute solvation energy. The calculations of solvent effects have been performed using water as solvent to simulate the internal environments of body.

Slovent cage effects was included according to the corrections proposed by Okuno [38], which take into account the free volume theory [39]. In this work the expression used to correct the Gibbs free energy is:

$$\Delta G_{\text{sol}}^{\text{FV}}≅\Delta G_{\text{sol}}^{0}-RT\left\{\ln\left[n10^{\left(2n-2\right)}\right]-(n-1)\right\}$$ (1)

where *n* represents the molecularity of the reaction. According to the Expression (1), the solvent cage effect causes a decrease of 10.63 kJ/mol in Δ*G*^0^ for a bimolecular reaction at 298.15 K.

For the mechanisms involving electronic transfers (ET), the Marcus theory [40–42] was used. Within the transition state formalism, the reaction energy barrier $\left(\Delta G_{\text{ET}}^{\neq }\right)$ were estimated in terms of the free energy of reaction $\left(\Delta G_{{}_{\text{ET}}}^{0}\right)$ and the nuclear reorganization energy (λ):

$$\Delta G_{\text{ET}}^{\neq }=\frac{\lambda }{4}{\left(1+\frac{\Delta G_{\text{ET}}^{0}}{\lambda }\right)}^{2}$$ (2)

reorganization energy (λ) is calculated as:

$$\lambda =\Delta E_{\text{ET}}-\Delta G_{\text{ET}}^{0}$$ (3)

Where Δ*E*_ET_ is calculated as the non-adiabatic energy difference between reactants and products, in reactants geometries.

The proton exchange method is applied to predict the p*K*a of PIC and ISO. We chose cathecol and guaiacol as the reference compounds (HRef), as which structures are similar with that of PIC and ISO, respectively. The calculation of pKa is based on the reaction scheme:

$$\text{ArOH}+{\text{Ref}}^{\text{-}}↔{\text{ArO}}^{\text{-}}+\text{HRef}$$ (4)

p*K*a is calculated as:

$$\text{p}K_{\text{a}}=\frac{\Delta G^{0}}{RT\ln\left(10\right)}+\text{p}K_{\text{a}}\left(\text{HRef}\right)$$ (5)

Where the experimental p*K*a values of catechol and Guaiacol are 9.25 [43] and 9.80 [44], respectively.

Four mechanisms of stilbene antioxidant (ArOH) scavenging radical (·R) were considered: hydrogen atom transfer (HAT):

$$\text{ArOH}+{\text{R}}^{\cdot }\to {\text{ArO}}^{\cdot }+\text{HR}$$ (6)

radical adduct formation (RAF):

$$\text{ArOH}+{\text{R}}^{\cdot }\to {\left(\text{ArOH}-\text{R}\right)}^{\cdot }$$ (7)

single electron transfer (SET):

$$\text{ArOH}+{\text{R}}^{\cdot }\to {\text{ArOH}}^{{+}^{\cdot }}+{\text{R}}^{\text{-}}$$ (8a)

$${\text{ArOH}}^{{+}^{\cdot }}+{\text{R}}^{\text{-}}\to {\text{ArO}}^{.}+\text{HR}$$ (8b)

sequential proton loss electron transfer (SPLET):

$$\text{ArOH}\to {\text{ArO}}^{-}+{\text{H}}^{+}$$ (9a)

$${\text{ArO}}^{\text{-}}+{\text{R}}^{\cdot }\to {\text{ArO}}^{\cdot }+{\text{R}}^{\text{-}}$$ (9b)

$${\text{H}}^{+}+{\text{R}}^{\text{-}}\to \text{HR}$$ (9c)

The products of ArO· and ArOH-R· are aromatic structures with the odd electron spreading over the entire molecule, resulting in a significant radical stabilization.

## Results and Discussion

**Fig 1:**
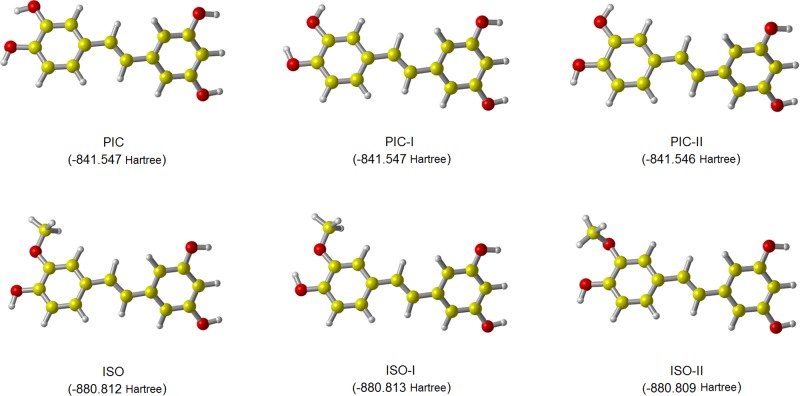
The geometries and energies of PIC’s and ISO’s conformers. The structures were fully optimized at M05-2X/6-311++G(d,p) level of theory, in water solution.

At the M05-2X/6-311++G(d,p) level, the optimized conformers of piceatannol and isorhapontigenin are showed in Fig 1, together with the energies of them. Among the conformers of piceatannol, PIC and PIC-I have relative lower and similar energies in water solution. While among the conformers of isorhapontigenin, ISO and ISO-I have relative lower and similar energies. According to the bond dissociation energy (BDE) obtained by Lu etc., PIC and ISO have lower BDE values and thus have higher reactivity than PIC-I and ISO-I, respectively [32]. Therefore, in this work, PIC and ISO were chosen out from their conformers.

The p*K*a value of a molecule determines the amount of protonated and nonprotonated forms at a specific pH. Therefore, the p*K*a values of PIC and ISO were calculated and reported in Table 1, together with the molar fractions of the neutral and anionic species in aqueous solution, at physiological pH (7.4). To identify which phenolic hydroxy group is involved in the deprotonation, all possible processes were first investigated. The Gibbs free energies of desprotonation in water solution are reported in Table 2, where calculated using Δ*G*_solv_(H^+^) = -265.9 kcal/mol in water solution based on the recommendation of Camaioni etc [45]. It was found that the most favourable anions of PIC and ISO are the products of deprotonation from A4- and A'4-OH groups, respectively. Therefore, the anion used in this work, when necessary, are deprotonation at these sites. According to the molar fractions, in water (at physiological PH), the dominant forms of PIC and ISO are neutral species, the proportions are 81.7% and 62.9%, respectively.

**Table 1:** p*K*_a_ values of PIC and ISO, and molar fractions (f) in water solution at PH = 7.4.

Piceatannol		Isorhapontigenin	
p*K*_a_	5.69	p*K*_a_	7.63
*f* (PIC)	0.8170	*f* (ISO)	0.6293
*f* (PIC^-^)	0.1830	*f* (ISO^-^)	0.3707

**Table 2:** The reaction Gibbs free energies (Δ*G*^0^) for all possible deprotonation sites of PIC and ISO in water solution (in kJ/mol).

PIC		ISO	
site	Δ*G*^0^	site	Δ*G*^0^
A4	37.68	A'4	47.97
A5	57.60		
B3	56.23	B'3	56.48
B5	55.32	B'5	51.71

In the light of the optimized geometries of PIC and ISO in Fig 1, both PIC and ISO have an approximately planar structure. At the M05-2X/6-311++G(d,p) level, the dihedral angle between two benzene rings of PIC is 179.95^。^, which is reasonably consistent with the experimental value of 179.23° [29]. At the same level, the obtained dihedral angle between two benzene rings of ISO is 179.90°.

### Nitric oxide radical (NO)

Four reaction mechanisms of PIC and ISO scavenging NO in water solution have been considered: HAT, RAF, SET and SPLET. For HAT mechanism, NO abstracts H atom from the hydroxyl group (‒OH) of PIC/ISO, followed by forming a water molecule and a semiquinone PIC/ISO radical. Therefore, we considered four HAT reaction channels (from sites A4, A5, B3 and B5) for PIC as well as three HAT channels (from sites A'4, B'3 and B'5) for ISO. As far as RAF mechanism, NO adds to either C atom of the carbon-carbon double bond which connects two benzene rings, so we considered two RAF channels (on sites C20 and C21) for PIC as well as two RAF channels (on sites C'20 and C'21) for ISO.

**Fig 2:**
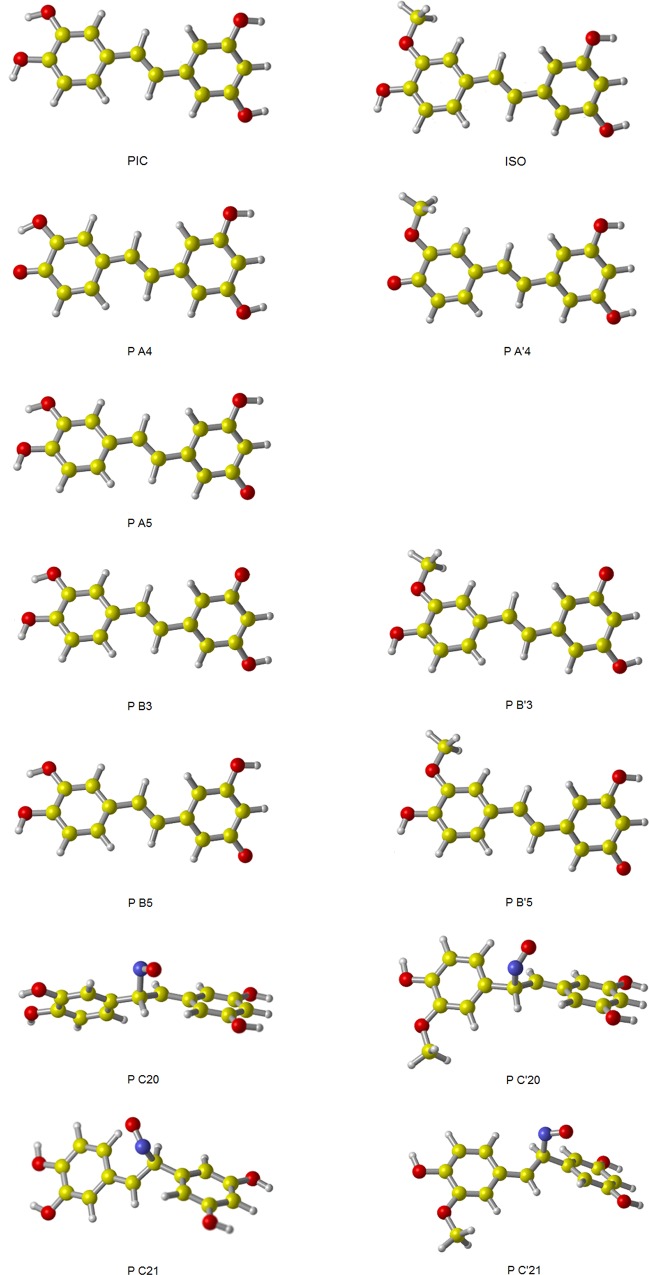
The geometries of reactants and products for reactions of PIC and ISO with NO. The structures were fully optimized at M05-2X/6-311++G(d,p) level of theory, in water solution.

**Fig 3:**
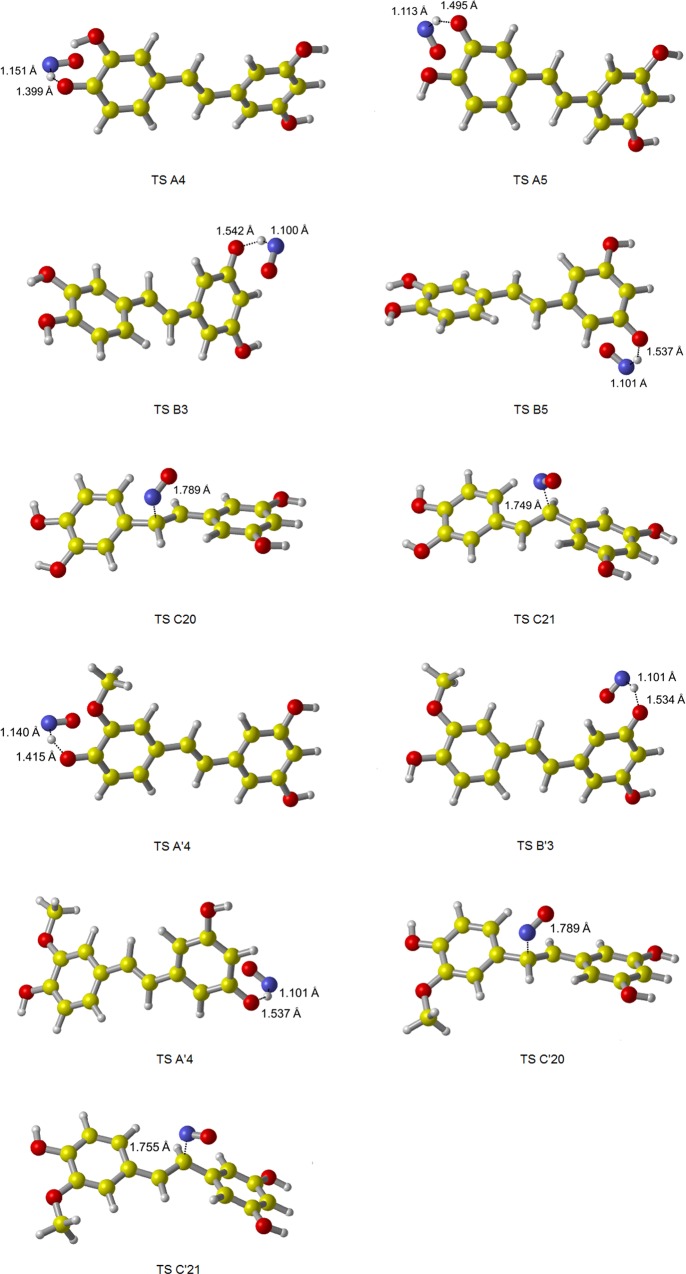
The geometries of TS for PIC and ISO scavenging NO, fully optimized at M05-2X/6-311++G(d,p) level of theory. All TS have only one imaginary frequency which corresponds to the expected motion along the reaction coordinate.

Under the M05-2X/6-311++G(d,p) level, the optimized geometries of all reactants and products for reactions of PIC and ISO with NO are shown in Fig 2, the geometries of TS for HAT and RAF mechanisms are shown in Fig 3. The Gibbs free energy of reaction (Δ*G*^0^*)* in water solution for HAT, RAF, SET and SPLET mechanisms were obtained and gathered in Table 3. It is can be seen, for both PIC and ISO, all channels are very endergonic. Apparently, the reactions of PIC and ISO with NO are less thermodynamical reactivity. This is agreed with the conclusion obtained by Alvarez-Idaboy and his coworkers, that NO itself might not react with any amino acid or even any antioxidant via it is an extremely poor acceptor of both H atoms and electrons, the importance of reaction involved NO is that it is a precursor of other RNS: NO_2_ and ONOO- [46]. In addition, it is can be found from Table 3 that the endergonic amount of SET mechanism is largest, reached 320.28 kJ/mol for PIC and 310.28 kJ/mol for ISO, indicating that SET mechanism seems irrelevant for PIC and ISO scavenging NO.

**Table 3:** The Gibbs free energies of reaction (Δ*G*^0^) and reaction energy barrier (Δ*G*^*≠*^) for PIC and ISO scavenging NO in water solution, at 298 K (in kJ/mol).

	PIC+NO			ISO+NO	
Mechanism	Δ*G*^0^	Δ*G*^*≠*^	Mechanism	Δ*G*^0^	Δ*G*^*≠*^
**HAT-A4**	97.60	125.37	**HAT-A'4**	103.48	121.41
**HAT-A5**	129.02	141.08			
**HAT-B3**	251.19	159.17	**HAT-B'3**	251.47	158.30
**HAT-B5**	253.79	158.60	**HAT-B'5**	250.51	158.39
**RAF-C20**	100.92	80.68	**RAF-C'20**	95.85	80.49
**RAF-C21**	94.60	70.15	**RAF-C'21**	96.10	64.73
**SET**	320.28	533.76	**SET**	310.28	483.82
**SPLET**	223.97	306.39	**SPLET**	214.82	286.98

The energy barriers (Δ*G*^*≠*^) of four mechanisms for PIC and ISO scavenging NO in water solution were obtained and listed in Table 3. As the electron transfer process occurs without passing through a saddle point, the Δ*G*^≠^ values for SET mechanism were calculated in terms of Eq (2). For SPLET mechanism, the the Δ*G*^≠^ values for step (9b) have also been calculated according to Eq (2). As once the PIC^-^ is formed at the proper pH conditions, the energy cost for the rest of the reaction to proceed is associated to the electron transfer (9b) from this species.

In Table 3, the barrier heights of SET mechanism are extremely high for both of PIC (533.76 kJ/mol) and ISO (483.82 kJ/mol), further confirmed that SET mechanism is irrelevant to the NO scavenging activity of PIC and ISO. Besides, the barrier heights of SPLET reactions of PIC (306.39 kJ/mol) and ISO (286.98 kJ/mol) are also much higher than that of HAT and RAF mechanisms, thus HAT and RAF are relative feasible mechanisms in kinetics.

**Fig 4:**
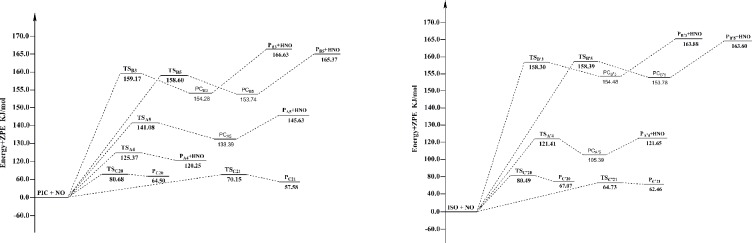
The potential energy surface profiles for HAT and RAF reactions of PIC and ISO with NO. The relative energies (in kJ/mol) were calculated at the M05-2X/6-311++G(d,p) + ZPE level. To facilitate the comparison, the energy of the reactants are set to zero.

The potential energy surface profiles for HAT and RAF channels of PIC and ISO are gathered in Fig 4, showing the relative energies of TS to reactants, and products to reactants. From Fig 4, it is easily to find that RAF-C21 channel has the lowest barrier height among all channels of PIC with NO, and RAF-C'21 channel has the lowest barrier height among all channels of ISO with NO. In addition, the barrier heights of all RAF channels are much lower than that of HAT channels for both PIC and ISO. Thus, we can conclude that RAF is the main mechanism of PIC and ISO scavenging NO, and the most efficient sites are C21 and C'21 atom on the carbon-carbon double bond. The barrier heights of channel RAF-C'21 (64.73 kJ/mol) are lower than that of channel RAF-C21 (70.15 kJ/mol in water), proved that ISO is more reactive than PIC in scavenging NO.

As far as the HAT process is concerned, the activity order in terms of barrier height is HAT-A4 (125.37 kJ/mol) > HAT-A5 (141.08 kJ/mol) > HAT-B5 (158.60 kJ/mol) > HAT-B3 (159.17 kJ/mol) for PIC with NO; HAT-A'4 (121.41 kJ/mol) > HAT-B'3 (158.30 kJ/mol) > HAT-B'5 (158.39 kJ/mol) for ISO with NO. We can find the reactivity of sites from A-ring are higher than that of from B-ring, showing that A-ring is more efficient for PIC and ISO scavenging NO. Among HAT mechanism, the most reactive sites are A4‒ and A'4‒OH groups, both of them have another ‒OH group on their ortho-positions (A5 and A'5). Therefore, it is avail to state that the existence of the adjacent ‒OH group may have a promoting effect on the radical scavenging activity of ‒OH group in PIC and ISO.

### Nitrogen dioxide radical (NO_2_)

Similar with scavenging NO, the HAT, RAF, SET and SPLET mechanisms of PIC and ISO scavenging NO_2_ in water solution were investigated. Four HAT channels (A4, A5, B3 and B5) and two RAF channels (C20 and C21) for PIC with NO_2_ were denoted, together with three HAT channels (A'4, B'3 and B'5) and two RAF channels (C'20 and C'21) for ISO with NO_2_.

**Fig 5:**
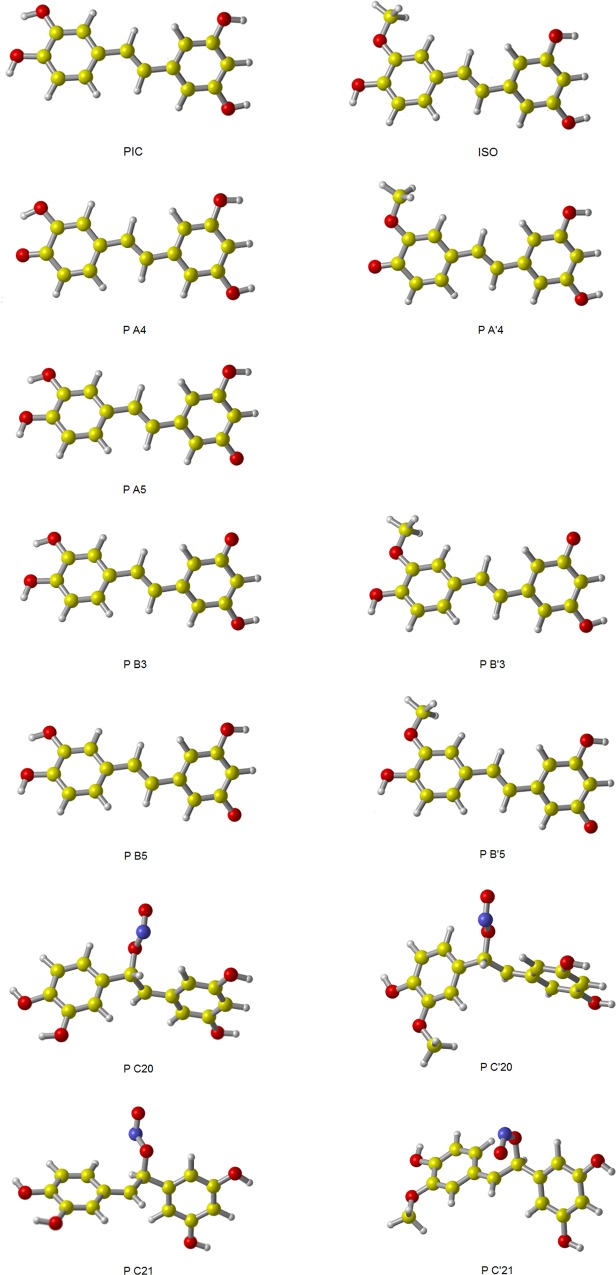
The geometries of reactants and products for reactions of PIC and ISO with NO_2_. The structures were fully optimized at M05-2X/6-311++G(d,p) level of theory, in water solution.

**Fig 6:**
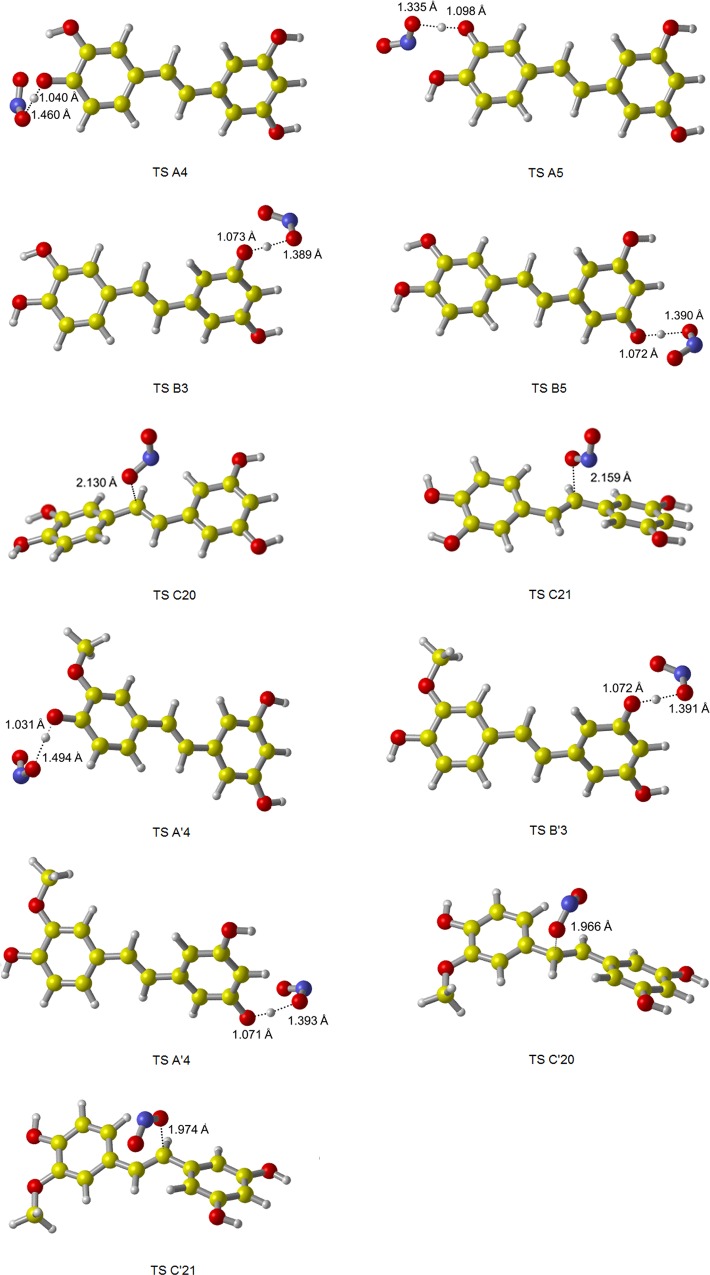
The geometries of TS for PIC and ISO scavenging NO_2_, fully optimized at M05-2X/6-311++G(d,p) level of theory. All TS have only one imaginary frequency which corresponds to the expected motion along the reaction coordinate.

The optimized geometries of all reactants and products for reactions of PIC and ISO with NO_2_ are shown in Fig 5, and the geometries of TS for HAT and RAF mechanisms are collected in Fig 6. The calculation results of Δ*G*^0^, Δ*G*^*≠*^ in water solution for four mechanisms are listed in Table 4. According to Table 4, HAT, SET and SPLET mechanisms are thermodynamically feasible for both PIC and ISO. Among them, SPLET is the most exergonic mechanism for PIC (-99.90 kJ/mol) and ISO (-109.05 kJ/mol). Thus, SPLET mechanism is most thermodynamically feasible for PIC and ISO scavenging NO_2_ in water solution.

**Table 4:** The Gibbs free energies of reaction (Δ*G*^0^) and reaction energy barrier (Δ*G*^*≠*^) for PIC and ISO scavenging NO_2_ in water solution, at 298 K (in kJ/mol).

	PIC+NO_2_			ISO+NO_2_	
Mechanism	Δ*G*^0^	Δ*G*^*≠*^	Mechanism	Δ*G*^0^	Δ*G*^*≠*^
**HAT-A4**	-47.78	-113.27	**HAT-A'4**	-41.90	34.76
**HAT-A5**	-16.36	-9.81			
**HAT-B3**	105.80	73.00	**HAT-B'3**	106.09	-21.08
**HAT-B5**	108.41	70.00	**HAT-B'5**	105.13	70.54
**RAF-C20**	20.84	28.87	**RAF-C'20**	19.00	7.44
**RAF-C21**	17.83	22.79	**RAF-C'21**	20.01	4.85
**SET**	-3.59	31.11	**SET**	-12.87	28.29
**SPLET**	-99.90	1.78	**SPLET**	-109.05	0.90

In terms of the energy barrier values, the channel HAT-A4 has the lowest barrier height among all reactions of PIC with NO_2_, and thus it is the main channel of total reaction. The channel HAT-B'3 has the lowest barrier height among all reactions of ISO with NO_2_, therefore HAT-B'3 is the kinetic superiority channel. In summary, HAT is the main mechanism of PIC and ISO scavenging NO_2_.

**Fig 7:**
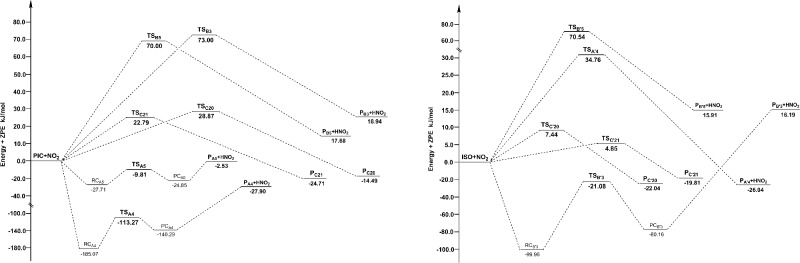
The potential energy surface profiles for HAT and RAF reactions of PIC and ISO with NO_2_. The relative energies (in kJ/mol) were calculated at the M05-2X/6-311++G(d,p) + ZPE level. To facilitate the comparison, the energy of the reactants are set to zero.

The potential energy surface profiles for HAT and RAF channels for PIC and ISO are shown in Fig 7. Making comparison of the activity between PIC and ISO, Δ*G*^*≠*^ value of the major channel of PIC (HAT-A4, -113.27 kJ/mol) is much lower than that of ISO (HAT-B'3, -21.08 kJ/mol). Moreover, the exergonic amounts of the channel HAT-A4 (-47.78 kJ/mol) are higher than that of the channel HAT-B'3 (-41.90 kJ/mol). From a general view, PIC is much efficient than ISO in scavenging NO_2_ in vivo. It is probably because the two adjacent ‒OH groups of PIC can form a intramolecular hydrogen bond after the H atom have been abstracted, which increases the stability of the product.

The above results are consistent with the previous theoretical study [47] on the reactions of *β*-carotene with NO_2_, they discovered that the ET reaction is thermodynamically favorable in polar solvents. RAF reaction was endergonic in water solution. The reaction mechanism is controlled by HAT and the lowest energy barrier in water solution is 22.18 kJ/mol. Hence, it seems that the NO_2_ scavenging capacity of PIC and ISO are better than *β*-carotene.

### Features of scavenging radicals

In the previous study, the radical scavenging activity of PIC and ISO toward ·OH and ·OOH were theoretically investigated using the same method of M05-2X/6-311++G(d,p), and the reaction energy barriers with ZPE corrections for HAT and RAF mechanisms were also obtained [32]. We gathered Δ*G*^*≠*^_sol_ values of the major channels in water solution of PIC and ISO scavenging ·OH, ·OOH, NO and NO_2_ in Table 5, in order to research the features of PIC and ISO scavenging radicals in vivo.

**Table 5:** The reaction energy barriers (Δ*G*^*≠*^) for the reactions of PIC and ISO scavenging ·OH, ·OOH, NO and NO_2_ in water solution (in kJ/mol).

	PIC		ISO	
radical	major channel	Δ*G*^*≠*^	major channel	Δ*G*^*≠*^
**·OH**	A4	-58.86	A'4	-56.45
**·OOH**	A5	30.36	A'5	15.70
**NO**	C21	70.15	C'21	64.73
**NO**_**2**_	A4	-113.27	B'3	-21.08

Table 5 shows that except scavenging NO, PIC and ISO scavenging other radicals (·OH, ·OOH and NO_2_) are mainly through HAT mechanism. While for scavenging NO, RAF is the main mechanism for both PIC and ISO. A-ring is the most efficient part of PIC scavenging most of radicals studied here. As far as ISO, the most reactive site is differ to different radical, covered several parts including A'-ring, B'-ring, and C' atom of carbon-carbon double bond.

In addition, in vivo environment, PIC and ISO are more sensitive to ·OH and NO_2_ via the energy barriers of them are relative lower. This is reasonable, since the activity of these radicals are different. However, it is interestingly to find that for scavenging radicals ·OH and NO_2_, PIC is more efficient than ISO; while for radicals ·OOH and NO, ISO is more efficient.

## Conclusions

For NO scavenging activity of PIC and ISO, RAF mechanism is favored with respect to HAT mechanism in water solution, while SET and SPLET are negligible mechanisms. The C atom linked with B/B'-ring is the most active site. For scavenging NO_2_, HAT reactions from sites A4 and B'3 are the major channels of PIC and ISO, respectively. Within the HAT processes of PIC scavenging NO and NO_2_, A4‒OH group in A-ring with an ortho‒OH group is more reactive, showing that the introducing of the adjacent ‒OH group could improve the radical scavenging activity of PIC.

Taking together four kinds of radicals ·OH, ·OOH, NO and NO_2_, A/A'-ring is the most important active position for PIC and ISO scavenging these radicals. In vivo, PIC is the best scavenger for NO_2_, ISO is the best scavenger for ·OH. Above results could provide some valuable theoretical data for designing and recognizing medical antioxidant.

## Supporting Information

S1 Table The geometry coordinates of all species optimized at M05-2X/6-311++G(d,p) level. (DOC)
