# Supplementary material for: A Theoretical Study on the Antioxidant Activity of Piceatannol and Isorhapontigenin Scavenging Nitric Oxide and Nitrogen Dioxide Radicals
Source: PLoS One. 2017 Jan 9;12(1):e0169773. doi: 10.1371/journal.pone.0169773 (PMC5222500; doi:10.1371/journal.pone.0169773)
Supplement: S1 Table — (DOC) [file pone.0169773.s001.doc]

Table S1. The geometry coordinates of all species optimized at M05-2X/6-311++G(d,p) level

Cartesian coordinates of the reactants in water

Piceatannol

C    -4.327115000000     -1.047018000000      0.038102000000
C    -2.962121000000     -1.307227000000      0.026894000000
C    -2.051582000000     -0.248232000000     -0.011060000000
C    -2.525711000000      1.067932000000     -0.041816000000
C    -3.892119000000      1.299125000000     -0.032566000000
C    -4.811760000000      0.253737000000      0.006706000000
H    -2.615541000000     -2.331567000000      0.051652000000
H    -1.857404000000      1.916080000000     -0.075337000000
H    -5.876878000000      0.451995000000      0.014346000000
C     1.818256000000     -0.025442000000     -0.010940000000
C     2.312742000000     -1.332984000000     -0.066141000000
C     2.732476000000      1.033290000000      0.041772000000
C     3.679804000000     -1.570239000000     -0.068481000000
H     1.638821000000     -2.176488000000     -0.109591000000
C     4.095661000000      0.796791000000      0.041340000000
H     2.382879000000      2.056960000000      0.086117000000
C     4.575038000000     -0.511056000000     -0.016597000000
H     4.066828000000     -2.580502000000     -0.112825000000
H     0.169656000000      1.362856000000      0.010921000000
C     0.387048000000      0.300753000000     -0.006268000000
C    -0.620309000000     -0.581441000000     -0.016696000000
H    -0.406764000000     -1.644061000000     -0.024942000000
O    -4.310539000000      2.606442000000     -0.065376000000
H    -5.273578000000      2.644647000000     -0.057496000000
O    -5.174020000000     -2.125935000000      0.081850000000
H    -6.089857000000     -1.825718000000      0.093796000000
O     4.965161000000      1.857193000000      0.097997000000
H     5.870052000000      1.521385000000      0.095154000000
O     5.938730000000     -0.659058000000     -0.015927000000
H     6.175274000000     -1.592669000000     -0.065640000000


Isorhapontigenin

C    -4.635218000000     -0.905153000000      0.361678000000
C    -3.295530000000     -1.268176000000      0.305213000000
C    -2.334793000000     -0.320405000000     -0.055969000000
C    -2.734517000000      0.980382000000     -0.379955000000
C    -4.078264000000      1.314819000000     -0.317608000000
C    -5.046219000000      0.384766000000      0.051703000000
H    -3.006250000000     -2.280493000000      0.553261000000
H    -2.025221000000      1.732162000000     -0.694955000000
H    -6.093038000000      0.661135000000      0.092008000000
C     1.534558000000     -0.311435000000     -0.150857000000
C     1.953758000000     -1.611512000000     -0.430978000000
C     2.503368000000      0.670637000000      0.108683000000
C     3.307324000000     -1.925386000000     -0.436148000000
H     1.235205000000     -2.385750000000     -0.659853000000
C     3.854139000000      0.359656000000      0.107356000000
H     2.178806000000      1.679989000000      0.317056000000
C     4.257389000000     -0.953633000000     -0.167921000000
H     3.639441000000     -2.932131000000     -0.657118000000
H    -0.045336000000      1.161714000000     -0.101927000000
C     0.123077000000      0.090326000000     -0.118408000000
C    -0.926374000000     -0.740256000000     -0.079561000000
H    -0.766112000000     -1.811634000000     -0.031857000000
O    -4.422357000000      2.603126000000     -0.644086000000
H    -5.377372000000      2.714567000000     -0.579590000000
O    -5.537509000000     -1.869503000000      0.735297000000
H    -6.426462000000     -1.497284000000      0.746979000000
O     4.857660000000      1.251767000000      0.352679000000
O     5.603196000000     -1.210737000000     -0.165558000000
H     5.750793000000     -2.137370000000     -0.387210000000
C     4.472996000000      2.589264000000      0.682464000000
H     3.932843000000      3.048873000000     -0.144946000000
H     5.399432000000      3.125938000000      0.858863000000
H     3.861182000000      2.599697000000      1.584469000000

NO

O     0.000000000000      0.000000000000      0.530223000000
N     0.000000000000      0.000000000000     -0.605969000000


NO2

N     0.000000000000      0.000000000000      0.314730000000
O     0.000000000000      1.089032000000     -0.137694000000
O     0.000000000000     -1.089032000000     -0.137694000000

Cartesian coordinates of the HAT transition structures for the reactions of PIC with NO:

HAT-A4

C    -4.811871000000     -1.177213000000      0.124826000000
C    -3.450492000000     -1.339516000000     -0.087842000000
C    -2.618972000000     -0.220655000000     -0.158239000000
C    -3.165362000000      1.059711000000     -0.036197000000
C    -4.526005000000      1.202829000000      0.174386000000
C    -5.362842000000      0.091620000000      0.260788000000
H    -3.052849000000     -2.339612000000     -0.184961000000
H    -2.565273000000      1.952642000000     -0.120179000000
H    -6.427513000000      0.216164000000      0.424220000000
C     1.205448000000      0.261184000000     -0.376883000000
C     1.735609000000     -0.925532000000     -0.948463000000
C     2.080364000000      1.283348000000      0.009562000000
C     3.083002000000     -1.093398000000     -1.099664000000
H     1.066747000000     -1.704019000000     -1.283399000000
C     3.441211000000      1.126775000000     -0.138842000000
H     1.700668000000      2.203228000000      0.432746000000
C     3.990277000000     -0.080662000000     -0.684254000000
H     3.498919000000     -1.989519000000     -1.538938000000
H    -0.493379000000      1.460766000000      0.159188000000
C    -0.219502000000      0.470878000000     -0.185263000000
C    -1.185622000000     -0.445539000000     -0.361381000000
H    -0.920329000000     -1.455832000000     -0.647358000000
O    -5.010101000000      2.471042000000      0.286622000000
H    -5.958986000000      2.453056000000      0.422373000000
O    -5.572466000000     -2.304475000000      0.193644000000
H    -6.492947000000     -2.074599000000      0.331664000000
O     4.299142000000      2.090310000000      0.223621000000
H     5.179164000000      1.790074000000     -0.048938000000
O     5.269788000000     -0.168778000000     -0.794971000000
H     5.746988000000     -0.838694000000      0.336728000000
O     4.514886000000     -1.133432000000      1.697814000000
N     5.648191000000     -1.227709000000      1.415399000000

HAT-A5

O     4.363481000000     -0.121438000000      2.065559000000
N     5.166402000000      0.737932000000      2.118365000000
C    -4.808857000000     -0.996532000000      0.503823000000
C    -3.461508000000     -1.272880000000      0.321191000000
C    -2.601337000000     -0.268577000000     -0.123111000000
C    -3.103967000000      1.003060000000     -0.405447000000
C    -4.451865000000      1.260751000000     -0.218537000000
C    -5.317668000000      0.269676000000      0.238975000000
H    -3.097263000000     -2.266115000000      0.541401000000
H    -2.478658000000      1.792530000000     -0.793367000000
H    -6.372347000000      0.480387000000      0.376665000000
C     1.241260000000     -0.016422000000     -0.492621000000
C     1.695733000000     -1.289217000000     -0.919612000000
C     2.176906000000      0.957945000000     -0.227436000000
C     3.044264000000     -1.567255000000     -1.052344000000
H     0.979305000000     -2.058767000000     -1.166175000000
C     3.578767000000      0.729057000000     -0.360552000000
H     1.879111000000      1.948899000000      0.088682000000
C     3.989732000000     -0.589926000000     -0.775644000000
H     3.366475000000     -2.548703000000     -1.378396000000
H    -0.410078000000      1.347489000000     -0.221344000000
C    -0.182942000000      0.294107000000     -0.336162000000
C    -1.178525000000     -0.596664000000     -0.283369000000
H    -0.955143000000     -1.656543000000     -0.320443000000
O    -4.894115000000      2.516212000000     -0.510047000000
H    -5.839079000000      2.576899000000     -0.360466000000
O    -5.600226000000     -2.012089000000      0.949048000000
H    -6.504600000000     -1.708715000000      1.045074000000
O     4.440301000000      1.633521000000     -0.138521000000
H     5.063768000000      1.339607000000      1.187733000000
O     5.314200000000     -0.792660000000     -0.858424000000
H     5.494613000000     -1.703614000000     -1.103757000000


HAT-B3

C    -3.723768000000      1.653608000000     -0.390330000000
C    -2.344285000000      1.732417000000     -0.116542000000
C    -1.529551000000      0.595167000000     -0.153174000000
C    -2.114158000000     -0.630950000000     -0.434318000000
C    -3.514875000000     -0.736040000000     -0.714223000000
C    -4.311802000000      0.454787000000     -0.690281000000
H    -1.927338000000      2.703308000000      0.112358000000
H    -1.548377000000     -1.551175000000     -0.425786000000
H    -5.368201000000      0.363377000000     -0.906542000000
C     2.288193000000      0.018968000000     -0.001737000000
C     2.789183000000      0.804765000000      1.037601000000
C     3.187709000000     -0.681696000000     -0.811050000000
C     4.157974000000      0.906530000000      1.245234000000
H     2.114599000000      1.318736000000      1.706415000000
C     4.551883000000     -0.581875000000     -0.609336000000
H     2.834792000000     -1.308106000000     -1.619219000000
C     5.036802000000      0.221408000000      0.423138000000
H     4.544185000000      1.509991000000      2.057597000000
H     0.569499000000     -0.960922000000     -0.882636000000
C     0.852579000000     -0.107349000000     -0.276044000000
C    -0.094587000000      0.748807000000      0.119990000000
H     0.186341000000      1.651853000000      0.649542000000
O    -4.036134000000     -1.865198000000     -0.974172000000
H    -4.446937000000     -2.505283000000      0.367337000000
O    -4.402491000000      2.830573000000     -0.336473000000
H    -5.329742000000      2.683983000000     -0.535550000000
O     5.409196000000     -1.263198000000     -1.410320000000
H     6.308904000000     -1.077284000000     -1.123005000000
O     6.401523000000      0.251407000000      0.549338000000
H     6.663904000000      0.840926000000      1.258283000000
O    -3.955071000000     -1.516812000000      1.834126000000
N    -4.475086000000     -2.507838000000      1.466804000000


HAT-B5

C    -3.886451000000     -0.572448000000     -0.626401000000
C    -2.487254000000     -0.877523000000     -0.680014000000
C    -1.527180000000      0.108734000000     -0.532361000000
C    -1.949124000000      1.434611000000     -0.352821000000
C    -3.316325000000      1.757712000000     -0.295749000000
C    -4.278006000000      0.789899000000     -0.426101000000
H    -2.212835000000     -1.912324000000     -0.832481000000
H    -1.241031000000      2.246206000000     -0.276072000000
H    -5.336115000000      1.015560000000     -0.397140000000
C     2.326071000000      0.122505000000     -0.188690000000
C     2.820760000000     -0.868864000000     -1.038309000000
C     3.218335000000      0.797403000000      0.649892000000
C     4.170399000000     -1.192798000000     -1.029418000000
H     2.162438000000     -1.375326000000     -1.728623000000
C     4.563184000000      0.477045000000      0.663549000000
H     2.871934000000      1.576975000000      1.315020000000
C     5.038272000000     -0.527617000000     -0.179840000000
H     4.553179000000     -1.957098000000     -1.694808000000
H     0.696214000000      1.448540000000      0.321223000000
C     0.906747000000      0.491165000000     -0.142523000000
C    -0.108017000000     -0.262987000000     -0.576975000000
H     0.092454000000     -1.254489000000     -0.965665000000
O    -3.603197000000      3.075321000000     -0.117224000000
H    -4.553852000000      3.203289000000     -0.092162000000
O    -4.754580000000     -1.489626000000     -0.762802000000
H    -5.026015000000     -2.081131000000      0.629873000000
O     5.410782000000      1.138023000000      1.491558000000
H     6.298302000000      0.787618000000      1.364364000000
O     6.385104000000     -0.770523000000     -0.100502000000
H     6.640841000000     -1.464504000000     -0.710354000000
O    -3.966305000000     -1.360840000000      1.944821000000
N    -4.809776000000     -2.147597000000      1.707797000000


Cartesian coordinates of the RAF transition structures for the reactions of PIC with NO:

C20

C     4.221517000000      1.216804000000     -0.464238000000
C     2.859139000000      1.418196000000     -0.353673000000
C     1.995032000000      0.320618000000     -0.182592000000
C     2.540285000000     -0.975388000000     -0.117308000000
C     3.906224000000     -1.151874000000     -0.231333000000
C     4.763495000000     -0.066210000000     -0.406832000000
H     2.471330000000      2.425238000000     -0.404582000000
H     1.925985000000     -1.851254000000      0.026837000000
H     5.833553000000     -0.217904000000     -0.492729000000
C    -1.824914000000     -0.082596000000      0.032370000000
C    -2.307129000000      1.034536000000      0.711201000000
C    -2.715846000000     -0.883695000000     -0.681043000000
C    -3.656818000000      1.363328000000      0.653293000000
H    -1.633847000000      1.647446000000      1.295642000000
C    -4.059722000000     -0.560492000000     -0.741961000000
H    -2.375134000000     -1.763044000000     -1.210514000000
C    -4.527848000000      0.571085000000     -0.072374000000
H    -4.032781000000      2.231220000000      1.181096000000
H    -0.176086000000     -1.412021000000     -0.353844000000
C    -0.386254000000     -0.455450000000      0.119272000000
C     0.590389000000      0.562353000000     -0.078300000000
H     0.268307000000      1.594369000000     -0.061104000000
O     4.379956000000     -2.428809000000     -0.163018000000
H     5.335614000000     -2.430352000000     -0.238075000000
O     5.009310000000      2.317660000000     -0.630762000000
H     5.928533000000      2.055983000000     -0.703590000000
O    -4.914437000000     -1.340201000000     -1.450157000000
H    -5.796538000000     -0.959373000000     -1.388888000000
O    -5.874563000000      0.804573000000     -0.192950000000
H    -6.120030000000      1.612549000000      0.260606000000
O     0.427150000000     -0.518455000000      2.484738000000
N    -0.319077000000     -1.068326000000      1.798207000000


C21

C    -4.134361000000     -0.981125000000     -0.775999000000
C    -2.803383000000     -1.269019000000     -0.513532000000
C    -1.962234000000     -0.262240000000     -0.044421000000
C    -2.458286000000      1.018484000000      0.184135000000
C    -3.793642000000      1.287468000000     -0.078716000000
C    -4.641198000000      0.296858000000     -0.564045000000
H    -2.439615000000     -2.271383000000     -0.688538000000
H    -1.842120000000      1.810391000000      0.582630000000
H    -5.682714000000      0.517006000000     -0.771437000000
C     1.869307000000      0.049147000000     -0.080013000000
C     2.386442000000     -1.217609000000      0.251584000000
C     2.776482000000      1.062399000000     -0.455925000000
C     3.750384000000     -1.455803000000      0.201181000000
H     1.729098000000     -2.009893000000      0.575122000000
C     4.130513000000      0.821645000000     -0.509789000000
H     2.419200000000      2.049739000000     -0.714478000000
C     4.620998000000     -0.447148000000     -0.179091000000
H     4.143442000000     -2.430197000000      0.464612000000
H     0.183578000000      1.375607000000     -0.221129000000
C     0.473622000000      0.348691000000     -0.048846000000
C    -0.553586000000     -0.595807000000      0.273578000000
H    -0.309094000000     -1.630769000000      0.057198000000
O    -4.233553000000      2.554567000000      0.160104000000
H    -5.172739000000      2.618150000000     -0.021404000000
O    -4.913437000000     -1.995042000000     -1.247748000000
H    -5.811216000000     -1.686737000000     -1.381865000000
O     4.986818000000      1.808036000000     -0.879759000000
H     5.883149000000      1.458171000000     -0.858495000000
O     5.981109000000     -0.586883000000     -0.261451000000
H     6.247791000000     -1.470322000000     -0.001373000000
O    -1.175512000000     -0.029616000000      2.596693000000
N    -0.354073000000     -0.610560000000      2.010836000000


Cartesian coordinates of the HAT transition structures for the reactions of ISO with NO:

HAT-A'4

C    -5.179079000000     -1.006567000000      0.072457000000
C    -3.831242000000     -1.312285000000     -0.049995000000
C    -2.879926000000     -0.290569000000     -0.048024000000
C    -3.294357000000      1.038995000000      0.076999000000
C    -4.642653000000      1.325674000000      0.197275000000
C    -5.599512000000      0.312098000000      0.197442000000
H    -3.539002000000     -2.348078000000     -0.146181000000
H    -2.599201000000      1.863873000000      0.082211000000
H    -6.653158000000      0.548963000000      0.293445000000
C     0.972441000000     -0.204479000000     -0.326721000000
C     1.408925000000     -1.545863000000     -0.443066000000
C     1.928826000000      0.826868000000     -0.339377000000
C     2.736517000000     -1.839265000000     -0.570794000000
H     0.689587000000     -2.350962000000     -0.427755000000
C     3.273299000000      0.549844000000     -0.475458000000
H     1.585899000000      1.845861000000     -0.236217000000
C     3.729695000000     -0.814830000000     -0.617485000000
H     3.087514000000     -2.857283000000     -0.665922000000
H    -0.603488000000      1.229777000000     -0.095709000000
C    -0.422753000000      0.165955000000     -0.191798000000
C    -1.473147000000     -0.673926000000     -0.180560000000
H    -1.304301000000     -1.738906000000     -0.277930000000
O    -4.993514000000      2.637242000000      0.314727000000
H    -5.945663000000      2.720107000000      0.389314000000
O    -6.058110000000     -2.046312000000      0.064467000000
H    -6.956161000000     -1.722485000000      0.153604000000
O     4.256496000000      1.461786000000     -0.470568000000
O     4.961410000000     -1.073855000000     -0.796983000000
H     5.627986000000     -0.907507000000      0.439754000000
C     3.896968000000      2.824109000000     -0.290166000000
H     3.253391000000      3.162306000000     -1.103519000000
H     4.828259000000      3.379614000000     -0.304487000000
H     3.395233000000      2.964985000000      0.668448000000
O     4.550964000000     -0.598421000000      1.917895000000
N     5.664448000000     -0.709763000000      1.561465000000


HAT-B'3

C     4.142428000000     -1.500884000000     -0.501730000000
C     2.751142000000     -1.722804000000     -0.513809000000
C     1.872313000000     -0.862148000000      0.153134000000
C     2.400873000000      0.207448000000      0.861100000000
C     3.810881000000      0.457600000000      0.880548000000
C     4.677074000000     -0.435354000000      0.170297000000
H     2.378094000000     -2.575758000000     -1.063597000000
H     1.778972000000      0.871337000000      1.443984000000
H     5.741709000000     -0.243995000000      0.202837000000
C    -1.968299000000     -0.462433000000      0.221666000000
C    -2.521455000000     -1.719244000000      0.438338000000
C    -2.826947000000      0.616988000000     -0.036769000000
C    -3.898513000000     -1.897483000000      0.372009000000
H    -1.889613000000     -2.559841000000      0.686362000000
C    -4.200127000000      0.445998000000     -0.107183000000
H    -2.394825000000      1.594253000000     -0.195488000000
C    -4.741602000000     -0.833925000000      0.098606000000
H    -4.327757000000     -2.876651000000      0.548962000000
H    -0.231275000000      0.819311000000      0.413866000000
C    -0.523898000000     -0.214203000000      0.260681000000
C     0.430631000000     -1.134058000000      0.085915000000
H     0.154450000000     -2.152118000000     -0.163820000000
O     4.281348000000      1.440696000000      1.534289000000
H     4.276949000000      2.664302000000      0.608344000000
O     4.888781000000     -2.404158000000     -1.191391000000
H     5.818633000000     -2.175100000000     -1.130170000000
O    -5.094332000000      1.429148000000     -0.361989000000
O    -6.092590000000     -0.953475000000      0.027054000000
H    -6.339720000000     -1.867488000000      0.179801000000
C    -4.585357000000      2.731487000000     -0.590124000000
H    -4.052613000000      3.101767000000      0.288061000000
H    -5.450546000000      3.357333000000     -0.782171000000
H    -3.923139000000      2.744487000000     -1.458085000000
O     3.469261000000      2.368261000000     -1.014690000000
N     3.989440000000      3.170353000000     -0.325877000000


HAT-B'5

C    -4.201484000000     -0.656932000000     -0.416436000000
C    -2.821871000000     -1.045371000000     -0.426499000000
C    -1.810954000000     -0.113419000000     -0.589483000000
C    -2.163161000000      1.232310000000     -0.772350000000
C    -3.509334000000      1.637513000000     -0.761963000000
C    -4.519942000000      0.728331000000     -0.588337000000
H    -2.603560000000     -2.096010000000     -0.293123000000
H    -1.415604000000      1.990393000000     -0.952633000000
H    -5.564098000000      1.013653000000     -0.590767000000
C     2.049353000000     -0.200203000000     -0.372318000000
C     2.477130000000     -1.383291000000     -0.964409000000
C     2.999277000000      0.622241000000      0.252524000000
C     3.817245000000     -1.747236000000     -0.908945000000
H     1.778586000000     -2.016313000000     -1.491741000000
C     4.336784000000      0.265257000000      0.312048000000
H     2.665825000000      1.545134000000      0.704043000000
C     4.748902000000     -0.941676000000     -0.276020000000
H     4.148821000000     -2.666721000000     -1.376645000000
H     0.489160000000      1.282797000000     -0.163707000000
C     0.648144000000      0.230905000000     -0.375927000000
C    -0.412526000000     -0.558860000000     -0.576356000000
H    -0.267162000000     -1.625131000000     -0.704061000000
O    -3.725609000000      2.967714000000     -0.948319000000
H    -4.667507000000      3.150846000000     -0.939134000000
O    -5.117513000000     -1.524246000000     -0.268694000000
H    -5.374464000000     -1.680606000000      1.238582000000
O     5.311721000000      0.997341000000      0.899644000000
O     6.069415000000     -1.249831000000     -0.199285000000
H     6.227856000000     -2.084518000000     -0.644345000000
C     4.938357000000      2.233057000000      1.482833000000
H     4.521259000000      2.907948000000      0.732610000000
H     5.851566000000      2.657084000000      1.886937000000
H     4.216540000000      2.082774000000      2.288110000000
O    -4.229935000000     -0.700557000000      2.288215000000
N    -5.132481000000     -1.457208000000      2.289032000000


Cartesian coordinates of the RAF transition structures for the reactions of ISO with NO:

RAF-C'20

C    -4.511997000000     -0.915338000000     -0.827229000000
C    -3.168927000000     -1.229775000000     -0.749388000000
C    -2.250750000000     -0.286830000000     -0.250211000000
C    -2.723606000000      0.969595000000      0.173872000000
C    -4.071693000000      1.261192000000      0.085845000000
C    -4.981799000000      0.330581000000     -0.414119000000
H    -2.837609000000     -2.203667000000     -1.079316000000
H    -2.066606000000      1.724506000000      0.578653000000
H    -6.037281000000      0.570408000000     -0.476390000000
C     1.574826000000     -0.208456000000      0.158532000000
C     1.973541000000     -1.496689000000      0.486146000000
C     2.537225000000      0.714922000000     -0.264356000000
C     3.310194000000     -1.866207000000      0.362902000000
H     1.251169000000     -2.217435000000      0.846181000000
C     3.870730000000      0.354391000000     -0.387370000000
H     2.222790000000      1.718481000000     -0.509422000000
C     4.258938000000     -0.958220000000     -0.070529000000
H     3.621173000000     -2.872374000000      0.617845000000
H     0.013672000000      1.276027000000      0.133714000000
C     0.155633000000      0.212727000000      0.314378000000
C    -0.867238000000     -0.640320000000     -0.189464000000
H    -0.605010000000     -1.653177000000     -0.461833000000
O    -4.474608000000      2.493448000000      0.509641000000
H    -5.424837000000      2.578799000000      0.417013000000
O    -5.354395000000     -1.866752000000     -1.322036000000
H    -6.254509000000     -1.537335000000     -1.327288000000
O     4.862151000000      1.179012000000     -0.797404000000
O     5.576443000000     -1.264543000000     -0.207090000000
H     5.718451000000     -2.174749000000      0.059323000000
C     4.512511000000      2.517613000000     -1.101754000000
H     3.803363000000      2.557076000000     -1.931026000000
H     5.437166000000      3.006544000000     -1.389820000000
H     4.089388000000      3.017362000000     -0.228193000000
O    -0.737109000000     -0.364955000000      2.580519000000
N     0.054757000000      0.320679000000      2.096856000000


RAF-C'21

C    -4.418722000000     -0.747028000000     -0.927689000000
C    -3.116137000000     -1.155735000000     -0.684905000000
C    -2.224359000000     -0.282025000000     -0.066346000000
C    -2.644484000000      0.985293000000      0.330000000000
C    -3.952201000000      1.376299000000      0.082828000000
C    -4.848412000000      0.521143000000     -0.550020000000
H    -2.812230000000     -2.145980000000     -0.992360000000
H    -1.990693000000      1.672292000000      0.845805000000
H    -5.867164000000      0.838017000000     -0.744389000000
C     1.616656000000     -0.234161000000     -0.006485000000
C     2.040493000000     -1.566115000000      0.117740000000
C     2.603359000000      0.763125000000     -0.203087000000
C     3.388397000000     -1.881458000000      0.038435000000
H     1.327213000000     -2.355115000000      0.301181000000
C     3.943127000000      0.447837000000     -0.287180000000
H     2.279536000000      1.789304000000     -0.293735000000
C     4.343515000000     -0.898302000000     -0.165193000000
H     3.709918000000     -2.911080000000      0.141691000000
H     0.030137000000      1.215122000000      0.047547000000
C     0.246631000000      0.155817000000      0.058732000000
C    -0.848342000000     -0.750699000000      0.220480000000
H    -0.666669000000     -1.758598000000     -0.137234000000
O    -4.316889000000      2.626159000000      0.485814000000
H    -5.247507000000      2.771045000000      0.306899000000
O    -5.248770000000     -1.632125000000     -1.548575000000
H    -6.122692000000     -1.250386000000     -1.646898000000
O     4.944011000000      1.338299000000     -0.480141000000
O     5.671949000000     -1.159138000000     -0.252137000000
H     5.818518000000     -2.100873000000     -0.143574000000
C     4.585651000000      2.703621000000     -0.597758000000
H     3.930182000000      2.861958000000     -1.456509000000
H     5.516203000000      3.241647000000     -0.744679000000
H     4.096143000000      3.057459000000      0.311808000000
O    -1.481480000000     -0.470041000000      2.597810000000
N    -0.691783000000     -1.025101000000      1.946425000000


Cartesian coordinates of the HAT transition structures for the reactions of PIC with NO2:

HAT-A4

C    -5.161772000000     -1.385923000000      0.142326000000
C    -3.789275000000     -1.437013000000     -0.054772000000
C    -3.050977000000     -0.253792000000     -0.114081000000
C    -3.699467000000      0.978395000000      0.003988000000
C    -5.070026000000      1.010541000000      0.197339000000
C    -5.814419000000     -0.165620000000      0.272522000000
H    -3.311025000000     -2.401384000000     -0.149588000000
H    -3.172184000000      1.916814000000     -0.071457000000
H    -6.887575000000     -0.128427000000      0.422907000000
C     0.725689000000      0.520438000000     -0.316858000000
C     1.344930000000     -0.627754000000     -0.868135000000
C     1.523363000000      1.599243000000      0.074760000000
C     2.707071000000     -0.689361000000     -1.024277000000
H     0.743590000000     -1.465342000000     -1.187478000000
C     2.896907000000      1.539956000000     -0.058664000000
H     1.082010000000      2.484406000000      0.511033000000
C     3.504316000000      0.397513000000     -0.634411000000
H     3.186218000000     -1.561818000000     -1.446593000000
H    -1.062457000000      1.597001000000      0.195178000000
C    -0.712722000000      0.625993000000     -0.134220000000
C    -1.603885000000     -0.364787000000     -0.303879000000
H    -1.258291000000     -1.354172000000     -0.576510000000
O    -5.656022000000      2.234635000000      0.303526000000
H    -6.602618000000      2.141634000000      0.424019000000
O    -5.828262000000     -2.570735000000      0.199954000000
H    -6.766309000000     -2.419380000000      0.328894000000
O     3.672112000000      2.553963000000      0.348056000000
H     4.594752000000      2.293286000000      0.221410000000
O     4.817920000000      0.448919000000     -0.774866000000
H     5.269920000000     -0.482614000000     -0.673685000000
N     5.999359000000     -1.749157000000      0.995473000000
O     5.777981000000     -1.774553000000     -0.221902000000
O     5.890028000000     -0.750090000000      1.638197000000


HAT-A5

C    -5.542376000000     -0.873483000000      0.440309000000
C    -4.204901000000     -1.216421000000      0.300822000000
C    -3.270113000000     -0.239208000000     -0.041800000000
C    -3.685979000000      1.074928000000     -0.266342000000
C    -5.024989000000      1.398799000000     -0.125538000000
C    -5.964994000000      0.434132000000      0.231882000000
H    -3.907902000000     -2.240851000000      0.473950000000
H    -3.001646000000      1.851518000000     -0.571567000000
H    -7.011328000000      0.698292000000      0.336541000000
C     0.587343000000     -0.198251000000     -0.275771000000
C     0.988973000000     -1.527006000000     -0.580301000000
C     1.561849000000      0.759137000000     -0.075809000000
C     2.319478000000     -1.861659000000     -0.661652000000
H     0.246215000000     -2.286802000000     -0.770595000000
C     2.934081000000      0.453011000000     -0.137649000000
H     1.300478000000      1.781183000000      0.161646000000
C     3.302144000000     -0.892062000000     -0.454543000000
H     2.617853000000     -2.874038000000     -0.903909000000
H    -0.987988000000      1.255267000000     -0.077232000000
C    -0.816553000000      0.190024000000     -0.173743000000
C    -1.864805000000     -0.643762000000     -0.152759000000
H    -1.702242000000     -1.714429000000     -0.184601000000
O    -5.381380000000      2.691647000000     -0.359114000000
H    -6.326373000000      2.802797000000     -0.241576000000
O    -6.408109000000     -1.865185000000      0.785678000000
H    -7.300654000000     -1.520313000000      0.847956000000
O     3.809459000000      1.368958000000      0.125379000000
H     4.878904000000      1.223757000000     -0.078585000000
O     4.611656000000     -1.136041000000     -0.539102000000
H     4.787741000000     -2.066059000000     -0.709740000000
N     6.453870000000      0.429011000000      0.827689000000
O     6.210996000000      1.173141000000     -0.150420000000
O     7.571742000000      0.134670000000      1.107736000000


HAT-B3

C    -3.112596000000      2.027549000000     -0.334099000000
C    -1.704868000000      2.048518000000     -0.339271000000
C    -0.963096000000      0.970227000000      0.155393000000
C    -1.647201000000     -0.124815000000      0.666562000000
C    -3.060514000000     -0.134332000000      0.692358000000
C    -3.800822000000      0.952422000000      0.172618000000
H    -1.211812000000      2.915043000000     -0.757045000000
H    -1.141001000000     -0.978411000000      1.090845000000
H    -4.882300000000      0.915917000000      0.184063000000
C     2.788520000000      0.044951000000      0.127358000000
C     3.510974000000      1.214279000000      0.372610000000
C     3.486162000000     -1.130704000000     -0.167250000000
C     4.896858000000      1.211376000000      0.300865000000
H     3.000146000000      2.126346000000      0.643983000000
C     4.866711000000     -1.138171000000     -0.241456000000
H     2.959266000000     -2.056781000000     -0.353691000000
C     5.572928000000      0.042697000000     -0.008823000000
H     5.455951000000      2.117833000000      0.498044000000
H     0.901110000000     -1.003052000000      0.235308000000
C     1.324181000000     -0.006509000000      0.171434000000
C     0.501407000000      1.045124000000      0.100103000000
H     0.910079000000      2.037564000000     -0.048546000000
O    -3.646694000000     -1.158725000000      1.246445000000
H    -4.679623000000     -1.245153000000      0.969805000000
O    -3.720669000000      3.119041000000     -0.853117000000
H    -4.674560000000      3.008122000000     -0.840654000000
O     5.524621000000     -2.286263000000     -0.536509000000
H     6.470367000000     -2.106332000000     -0.541259000000
O     6.935333000000     -0.063948000000     -0.099752000000
H     7.356619000000      0.773461000000      0.101208000000
N    -5.869907000000     -2.056167000000     -0.650963000000
O    -5.938171000000     -1.369725000000      0.395900000000
O    -4.820263000000     -2.521264000000     -0.982435000000


HAT-B5

C    -3.262661000000     -0.259176000000      0.671742000000
C    -1.926110000000     -0.709284000000      0.562608000000
C    -0.935696000000      0.134243000000      0.083228000000
C    -1.301451000000      1.413332000000     -0.358113000000
C    -2.634789000000      1.850884000000     -0.271697000000
C    -3.619320000000      1.037634000000      0.239244000000
H    -1.706232000000     -1.715072000000      0.890584000000
H    -0.582618000000      2.082478000000     -0.807280000000
H    -4.651844000000      1.355858000000      0.300915000000
C     2.920493000000     -0.007680000000     -0.098423000000
C     3.274743000000     -1.302241000000     -0.482286000000
C     3.931374000000      0.897296000000      0.239746000000
C     4.607548000000     -1.688094000000     -0.502634000000
H     2.517228000000     -2.007455000000     -0.791230000000
C     5.260184000000      0.516668000000      0.223668000000
H     3.693517000000      1.911198000000      0.532548000000
C     5.596584000000     -0.785800000000     -0.147292000000
H     4.882042000000     -2.690364000000     -0.808028000000
H     1.395861000000      1.523372000000     -0.019090000000
C     1.527869000000      0.446964000000     -0.040913000000
C     0.450447000000     -0.342178000000      0.027673000000
H     0.574144000000     -1.416457000000      0.094864000000
O    -2.875450000000      3.101347000000     -0.730164000000
H    -3.810750000000      3.308473000000     -0.663412000000
O    -4.131766000000     -1.067726000000      1.210304000000
H    -5.144111000000     -0.794619000000      0.988120000000
O     6.225349000000      1.406704000000      0.563015000000
H     7.081174000000      0.970266000000      0.501409000000
O     6.936637000000     -1.068451000000     -0.138602000000
H     7.095503000000     -1.973738000000     -0.411372000000
N    -6.615156000000     -1.112738000000     -0.575341000000
O    -6.394753000000     -0.467297000000      0.476276000000
O    -5.806655000000     -1.902101000000     -0.963324000000


Cartesian coordinates of the RAF transition structures for the reactions of PIC with NO2:

RAF-C20

C    -4.169388000000     -1.472916000000      0.068780000000
C    -2.806295000000     -1.603899000000      0.258603000000
C    -1.940853000000     -0.588377000000     -0.175499000000
C    -2.461059000000      0.554192000000     -0.798655000000
C    -3.829219000000      0.664413000000     -0.981739000000
C    -4.692204000000     -0.342213000000     -0.556204000000
H    -2.426043000000     -2.489415000000      0.746735000000
H    -1.836143000000      1.368130000000     -1.126661000000
H    -5.761329000000     -0.247551000000     -0.711178000000
C     1.886885000000     -0.166587000000     -0.126817000000
C     2.369914000000     -0.975177000000      0.901950000000
C     2.784677000000      0.407622000000     -1.030621000000
C     3.729932000000     -1.226680000000      1.008196000000
H     1.694009000000     -1.372218000000      1.645249000000
C     4.138559000000      0.148765000000     -0.935304000000
H     2.439422000000      1.056017000000     -1.824327000000
C     4.608531000000     -0.673324000000      0.091055000000
H     4.112060000000     -1.842844000000      1.812519000000
H     0.220535000000      0.971791000000     -0.900257000000
C     0.462458000000      0.126949000000     -0.270174000000
C    -0.529036000000     -0.771245000000      0.053458000000
H    -0.242765000000     -1.685568000000      0.557095000000
O    -4.285088000000      1.787336000000     -1.598415000000
H    -5.243586000000      1.793341000000     -1.619852000000
O    -4.966850000000     -2.485183000000      0.506555000000
H    -5.890002000000     -2.264879000000      0.370503000000
O     5.000722000000      0.695830000000     -1.826886000000
H     5.895743000000      0.431079000000     -1.590782000000
O     5.963279000000     -0.862945000000      0.108294000000
H     6.228375000000     -1.365066000000      0.881133000000
N    -0.741907000000      2.052205000000      1.372650000000
O     0.297904000000      1.336890000000      1.474664000000
O    -0.924258000000      2.877147000000      2.224424000000


RAF-C21

C     4.130118000000     -0.077160000000     -1.350414000000
C     2.781238000000      0.217758000000     -1.466342000000
C     1.902966000000     -0.176804000000     -0.455864000000
C     2.378168000000     -0.836865000000      0.677819000000
C     3.732184000000     -1.116880000000      0.778803000000
C     4.615226000000     -0.749080000000     -0.232174000000
H     2.430894000000      0.746040000000     -2.341162000000
H     1.733538000000     -1.094281000000      1.503890000000
H     5.672289000000     -0.974861000000     -0.144519000000
C    -1.927260000000     -0.305296000000     -0.177975000000
C    -2.399139000000      0.885179000000     -0.753234000000
C    -2.854905000000     -1.258639000000      0.277028000000
C    -3.759445000000      1.100799000000     -0.886187000000
H    -1.706113000000      1.658099000000     -1.048058000000
C    -4.208843000000     -1.049592000000      0.134314000000
H    -2.520717000000     -2.177797000000      0.738700000000
C    -4.660291000000      0.138195000000     -0.453868000000
H    -4.128236000000      2.024485000000     -1.314631000000
H    -0.259365000000     -1.390886000000      0.642675000000
C    -0.524070000000     -0.570532000000     -0.011979000000
C     0.487842000000      0.149383000000     -0.606811000000
H     0.245187000000      0.804890000000     -1.430327000000
O     4.153516000000     -1.760645000000      1.901023000000
H     5.108431000000     -1.846592000000      1.897453000000
O     4.947787000000      0.312838000000     -2.366602000000
H     5.856450000000      0.078563000000     -2.169862000000
O    -5.093468000000     -1.981973000000      0.563306000000
H    -5.986304000000     -1.663561000000      0.396175000000
O    -6.015393000000      0.259475000000     -0.545405000000
H    -6.259880000000      1.103651000000     -0.929862000000
N     0.968327000000      1.789453000000      1.632171000000
O     0.302883000000      1.948703000000      0.571834000000
O     1.063775000000      2.734512000000      2.367798000000


Cartesian coordinates of the HAT transition structures for the reactions of ISO with NO2:

HAT-A'4

C    -5.320681000000     -1.418253000000      0.211113000000
C    -3.947554000000     -1.511871000000      0.035382000000
C    -3.185124000000     -0.352534000000     -0.116563000000
C    -3.811130000000      0.897116000000     -0.112901000000
C    -5.182172000000      0.971556000000      0.061447000000
C    -5.950513000000     -0.179212000000      0.228630000000
H    -3.487588000000     -2.489499000000      0.030233000000
H    -3.266157000000      1.815885000000     -0.264485000000
H    -7.024196000000     -0.108926000000      0.362899000000
C     0.605180000000      0.342542000000     -0.327914000000
C     1.222160000000     -0.856690000000     -0.741983000000
C     1.412466000000      1.453235000000     -0.038241000000
C     2.586345000000     -0.940761000000     -0.855608000000
H     0.622693000000     -1.722051000000     -0.981415000000
C     2.790963000000      1.382019000000     -0.132044000000
H     0.935788000000      2.368056000000      0.280483000000
C     3.403475000000      0.167234000000     -0.560681000000
H     3.066018000000     -1.859528000000     -1.163945000000
H    -1.173891000000      1.483225000000      0.059099000000
C    -0.832068000000      0.482969000000     -0.178838000000
C    -1.738337000000     -0.505089000000     -0.275734000000
H    -1.406105000000     -1.519751000000     -0.456639000000
O    -5.745569000000      2.211365000000      0.055350000000
H    -6.695985000000      2.145114000000      0.162825000000
O    -6.011309000000     -2.580690000000      0.362059000000
H    -6.946660000000     -2.400391000000      0.471732000000
O     3.636389000000      2.382845000000      0.138921000000
O     4.706073000000      0.142920000000     -0.672662000000
H     5.075766000000     -0.819615000000     -0.690051000000
C     3.088508000000      3.613908000000      0.588994000000
H     2.436823000000      4.046015000000     -0.171932000000
H     3.937157000000      4.266451000000      0.760774000000
H     2.537361000000      3.470746000000      1.519513000000
N     5.655120000000     -2.429398000000      0.768276000000
O     5.530737000000     -1.544499000000      1.562117000000
O     5.502933000000     -2.230309000000     -0.446709000000


HAT-B'3

C     3.478989000000     -2.015304000000     -0.439348000000
C     2.075716000000     -2.125807000000     -0.424756000000
C     1.276049000000     -1.125666000000      0.140952000000
C     1.899647000000     -0.022461000000      0.708376000000
C     3.309187000000      0.076632000000      0.712348000000
C     4.107177000000     -0.927776000000      0.116905000000
H     1.630025000000     -2.996726000000     -0.884465000000
H     1.348349000000      0.769620000000      1.192126000000
H     5.183990000000     -0.820189000000      0.112264000000
C    -2.518378000000     -0.403509000000      0.180832000000
C    -3.184723000000     -1.610288000000      0.364549000000
C    -3.274549000000      0.756502000000     -0.048272000000
C    -4.571518000000     -1.658635000000      0.297198000000
H    -2.635441000000     -2.514341000000      0.583483000000
C    -4.657559000000      0.715625000000     -0.118067000000
H    -2.755197000000      1.693890000000     -0.182931000000
C    -5.313936000000     -0.514504000000      0.055043000000
H    -5.088835000000     -2.598698000000      0.447542000000
H    -0.684091000000      0.729553000000      0.322584000000
C    -1.059665000000     -0.282789000000      0.217364000000
C    -0.181496000000     -1.284409000000      0.093572000000
H    -0.535437000000     -2.290299000000     -0.097918000000
O     3.841121000000      1.102734000000      1.317065000000
H     4.853076000000      1.282084000000      1.012301000000
O     4.145169000000     -3.034710000000     -1.029527000000
H     5.090849000000     -2.867875000000     -1.017101000000
O    -5.456716000000      1.783495000000     -0.341350000000
O    -6.668756000000     -0.508949000000     -0.016240000000
H    -6.999036000000     -1.397570000000      0.129698000000
C    -4.830003000000      3.042350000000     -0.517834000000
H    -4.265556000000      3.324823000000      0.372976000000
H    -5.633798000000      3.752575000000     -0.680021000000
H    -4.170032000000      3.030520000000     -1.387451000000
N     5.906094000000      2.311709000000     -0.579870000000
O     6.071789000000      1.538180000000      0.392209000000
O     4.815999000000      2.744362000000     -0.809301000000


HAT-B'5

C    -3.632179000000     -0.548185000000     -0.501380000000
C    -2.286609000000     -0.976430000000     -0.421015000000
C    -1.247010000000     -0.067405000000     -0.540442000000
C    -1.563077000000      1.287834000000     -0.720127000000
C    -2.900114000000      1.716630000000     -0.765312000000
C    -3.939491000000      0.821806000000     -0.660743000000
H    -2.103696000000     -2.031002000000     -0.273111000000
H    -0.796839000000      2.039278000000     -0.837127000000
H    -4.974143000000      1.137336000000     -0.695571000000
C     2.606346000000     -0.256952000000     -0.269010000000
C     2.993350000000     -1.518928000000     -0.707282000000
C     3.584446000000      0.606002000000      0.249505000000
C     4.321159000000     -1.915125000000     -0.607958000000
H     2.273369000000     -2.192849000000     -1.148249000000
C     4.909781000000      0.216313000000      0.354417000000
H     3.282914000000      1.588209000000      0.582423000000
C     5.280840000000     -1.066996000000     -0.080540000000
H     4.621413000000     -2.896158000000     -0.956186000000
H     1.100437000000      1.288055000000     -0.207284000000
C     1.221718000000      0.216765000000     -0.325873000000
C     0.135426000000     -0.550728000000     -0.472614000000
H     0.245602000000     -1.627440000000     -0.518142000000
O    -3.087085000000      3.047940000000     -0.927329000000
H    -4.024594000000      3.255691000000     -0.935350000000
O    -4.563320000000     -1.459168000000     -0.452869000000
H    -5.525608000000     -1.054148000000     -0.213854000000
O     5.908917000000      0.981328000000      0.849948000000
O     6.589793000000     -1.404219000000      0.034322000000
H     6.718660000000     -2.300031000000     -0.283481000000
C     5.571445000000      2.276475000000      1.315280000000
H     5.170782000000      2.889854000000      0.505733000000
H     6.497369000000      2.710843000000      1.677062000000
H     4.848781000000      2.220833000000      2.131815000000
N    -6.676644000000     -0.509889000000      1.545899000000
O    -6.671902000000     -0.446771000000      0.293912000000
O    -5.793169000000     -1.082324000000      2.110591000000


Cartesian coordinates of the RAF transition structures for the reactions of ISO with NO2:

RAF-C'20

C    -4.514842000000     -0.680147000000     -1.078131000000
C    -3.182085000000     -1.048018000000     -1.008623000000
C    -2.263367000000     -0.212564000000     -0.360082000000
C    -2.699835000000      0.978570000000      0.236079000000
C    -4.037384000000      1.325002000000      0.157247000000
C    -4.954876000000      0.507970000000     -0.501378000000
H    -2.867633000000     -1.976264000000     -1.463097000000
H    -2.031604000000      1.619412000000      0.790961000000
H    -6.000362000000      0.790714000000     -0.556316000000
C     1.577782000000     -0.202109000000     -0.046767000000
C     1.986957000000     -1.525480000000      0.063430000000
C     2.533676000000      0.794490000000     -0.288922000000
C     3.330080000000     -1.846481000000     -0.086855000000
H     1.273214000000     -2.299650000000      0.302798000000
C     3.873136000000      0.478455000000     -0.445443000000
H     2.207181000000      1.821687000000     -0.357834000000
C     4.272995000000     -0.864696000000     -0.343896000000
H     3.655264000000     -2.875597000000      0.007575000000
H     0.000772000000      1.268926000000      0.052617000000
C     0.171642000000      0.201141000000      0.088246000000
C    -0.879233000000     -0.620917000000     -0.323811000000
H    -0.655595000000     -1.628346000000     -0.641175000000
O    -4.417618000000      2.490132000000      0.750658000000
H    -5.365664000000      2.609066000000      0.672470000000
O    -5.365703000000     -1.521440000000     -1.728391000000
H    -6.258042000000     -1.171607000000     -1.707664000000
O     4.860108000000      1.370395000000     -0.690732000000
O     5.594029000000     -1.128319000000     -0.499349000000
H     5.746627000000     -2.068935000000     -0.389048000000
C     4.496909000000      2.737308000000     -0.777224000000
H     3.798265000000      2.903819000000     -1.599630000000
H     5.418954000000      3.276020000000     -0.968037000000
H     4.056654000000      3.083499000000      0.159971000000
N    -0.350866000000     -0.661742000000      2.652473000000
O    -0.025257000000      0.375443000000      2.036945000000
O    -0.494471000000     -1.693173000000      2.059102000000


RAF-C'21

C    -4.446085000000     -0.672040000000     -1.098236000000
C    -3.130343000000     -1.080055000000     -0.930115000000
C    -2.238350000000     -0.254885000000     -0.249370000000
C    -2.660548000000      0.964622000000      0.277878000000
C    -3.978611000000      1.353847000000      0.100640000000
C    -4.880029000000      0.546472000000     -0.589865000000
H    -2.820945000000     -2.033321000000     -1.333560000000
H    -2.000387000000      1.590249000000      0.858621000000
H    -5.909121000000      0.861828000000     -0.722020000000
C     1.609243000000     -0.209529000000     -0.170169000000
C     2.024174000000     -1.533486000000      0.009358000000
C     2.589166000000      0.785447000000     -0.374262000000
C     3.372256000000     -1.851201000000     -0.040182000000
H     1.303306000000     -2.309614000000      0.219658000000
C     3.931880000000      0.470820000000     -0.429162000000
H     2.263964000000      1.808043000000     -0.494636000000
C     4.328201000000     -0.871164000000     -0.261899000000
H     3.694305000000     -2.875084000000      0.106639000000
H     0.013260000000      1.238858000000     -0.106580000000
C     0.228111000000      0.180258000000     -0.134127000000
C    -0.841691000000     -0.711709000000     -0.116422000000
H    -0.662215000000     -1.712805000000     -0.483730000000
O    -4.353761000000      2.550919000000      0.630495000000
H    -5.290641000000      2.696934000000      0.489010000000
O    -5.279104000000     -1.508350000000     -1.777575000000
H    -6.163352000000     -1.139360000000     -1.809796000000
O     4.934248000000      1.355013000000     -0.630693000000
O     5.654640000000     -1.132170000000     -0.318399000000
H     5.803564000000     -2.069065000000     -0.174264000000
C     4.577445000000      2.717936000000     -0.788387000000
H     3.935334000000      2.853123000000     -1.661015000000
H     5.510231000000      3.251742000000     -0.935287000000
H     4.074894000000      3.094060000000      0.104690000000
N    -0.582770000000     -0.769750000000      2.637417000000
O    -0.860756000000     -1.527067000000      1.680775000000
O    -0.417985000000      0.401736000000      2.446179000000


Cartesian coordinates of the HAT products in water:

HNO

N     0.061807000000      0.576078000000      0.000000000000
H    -0.927112000000      0.912588000000      0.000000000000
O     0.061807000000     -0.618142000000      0.000000000000

HNO2

N     0.000000000000      0.534083000000      0.000000000000
O     1.076444000000      0.058035000000      0.000000000000
O    -0.999543000000     -0.366943000000      0.000000000000
H    -0.615207000000     -1.267316000000      0.000000000000


Products for PIC

A4

C    -4.258371000000     -1.043451000000      0.000048000000
C    -2.894331000000     -1.304980000000      0.000014000000
C    -1.984309000000     -0.243224000000     -0.000040000000
C    -2.453435000000      1.076071000000     -0.000063000000
C    -3.819087000000      1.308112000000     -0.000039000000
C    -4.737544000000      0.260347000000      0.000006000000
H    -2.547025000000     -2.329167000000      0.000048000000
H    -1.784263000000      1.923711000000     -0.000098000000
H    -5.802513000000      0.460023000000      0.000021000000
C     1.854695000000     -0.010491000000     -0.000020000000
C     2.339862000000     -1.358713000000     -0.000090000000
C     2.779239000000      1.052554000000      0.000041000000
C     3.670031000000     -1.629208000000     -0.000080000000
H     1.638742000000     -2.179981000000     -0.000158000000
C     4.123648000000      0.795766000000      0.000060000000
H     2.428519000000      2.076054000000      0.000083000000
C     4.640365000000     -0.566534000000      0.000035000000
H     4.041585000000     -2.644628000000     -0.000151000000
H     0.233682000000      1.375543000000      0.000007000000
C     0.454590000000      0.315264000000     -0.000025000000
C    -0.562643000000     -0.578460000000     -0.000051000000
H    -0.351829000000     -1.640046000000     -0.000067000000
O    -4.236377000000      2.614241000000     -0.000075000000
H    -5.199414000000      2.655350000000      0.000033000000
O    -5.106565000000     -2.119876000000      0.000107000000
H    -6.022829000000     -1.820557000000      0.000362000000
O     5.030956000000      1.788441000000      0.000125000000
H     5.910410000000      1.375200000000      0.000146000000
O     5.879322000000     -0.744034000000     -0.000035000000


A5

C    -4.250849000000     -1.081403000000     -0.000239000000
C    -2.880310000000     -1.310937000000     -0.000166000000
C    -1.995655000000     -0.230263000000      0.000055000000
C    -2.495702000000      1.076087000000      0.000293000000
C    -3.867042000000      1.276697000000      0.000274000000
C    -4.762197000000      0.209312000000     -0.000060000000
H    -2.510632000000     -2.327315000000     -0.000238000000
H    -1.845402000000      1.938815000000      0.000577000000
H    -5.831252000000      0.384419000000     -0.000125000000
C     1.862048000000      0.070636000000     -0.000358000000
C     2.360391000000     -1.272669000000      0.000048000000
C     2.757562000000      1.108055000000     -0.000607000000
C     3.710832000000     -1.542191000000      0.000402000000
H     1.672089000000     -2.104058000000      0.000072000000
C     4.176621000000      0.891491000000     -0.000329000000
H     2.417536000000      2.135710000000     -0.000908000000
C     4.626426000000     -0.495826000000      0.000240000000
H     4.073228000000     -2.561814000000      0.000763000000
H     0.195600000000      1.429028000000     -0.001066000000
C     0.429563000000      0.371042000000     -0.000492000000
C    -0.558533000000     -0.533900000000      0.000154000000
H    -0.329005000000     -1.592356000000      0.000780000000
O    -4.314695000000      2.573776000000      0.000534000000
H    -5.278439000000      2.590958000000      0.001961000000
O    -5.073038000000     -2.178796000000     -0.000530000000
H    -5.995694000000     -1.899918000000      0.000540000000
O     5.010246000000      1.826925000000     -0.000525000000
O     5.949780000000     -0.671645000000      0.000625000000
H     6.174699000000     -1.612336000000      0.001527000000


B3

C     4.414085000000     -0.972371000000     -0.000190000000
C     3.017480000000     -1.227690000000     -0.000369000000
C     2.078002000000     -0.184055000000     -0.000005000000
C     2.548754000000      1.116601000000      0.000477000000
C     3.968224000000      1.401458000000      0.000346000000
C     4.897464000000      0.300743000000      0.000253000000
H     2.687324000000     -2.257551000000     -0.000838000000
H     1.887380000000      1.971032000000      0.000889000000
H     5.959068000000      0.508671000000      0.000384000000
C    -1.788368000000     -0.014872000000     -0.000202000000
C    -2.266797000000     -1.329414000000      0.000187000000
C    -2.713719000000      1.035277000000     -0.000451000000
C    -3.630693000000     -1.581964000000      0.000389000000
H    -1.582887000000     -2.165885000000      0.000349000000
C    -4.074056000000      0.783702000000     -0.000270000000
H    -2.375341000000      2.063666000000     -0.000773000000
C    -4.537850000000     -0.531313000000      0.000204000000
H    -4.007011000000     -2.597190000000      0.000735000000
H    -0.154871000000      1.391358000000     -0.000704000000
C    -0.361871000000      0.326891000000     -0.000299000000
C     0.654544000000     -0.544668000000      0.000067000000
H     0.458897000000     -1.610277000000      0.000391000000
O     4.367977000000      2.590195000000      0.000320000000
O     5.203434000000     -2.080646000000     -0.000423000000
H     6.134714000000     -1.827361000000     -0.000334000000
O    -4.954985000000      1.835679000000     -0.000509000000
H    -5.856597000000      1.491322000000     -0.000402000000
O    -5.898646000000     -0.695759000000      0.000436000000
H    -6.124117000000     -1.633478000000      0.000893000000


B5

C    -4.423829000000     -1.120170000000      0.000329000000
C    -2.997064000000     -1.366613000000      0.000243000000
C    -2.087212000000     -0.332225000000     -0.000126000000
C    -2.578142000000      0.992101000000     -0.000318000000
C    -3.968167000000      1.256401000000      0.000030000000
C    -4.883910000000      0.245011000000      0.000325000000
H    -2.670758000000     -2.398074000000      0.000453000000
H    -1.911781000000      1.841862000000     -0.000735000000
H    -5.947867000000      0.440988000000      0.000524000000
C     1.772993000000     -0.044431000000     -0.000570000000
C     2.287922000000     -1.344916000000     -0.000646000000
C     2.668622000000      1.031348000000     -0.000269000000
C     3.658471000000     -1.559052000000     -0.000396000000
H     1.628002000000     -2.200563000000     -0.000910000000
C     4.035354000000      0.817964000000      0.000082000000
H     2.301921000000      2.049899000000     -0.000144000000
C     4.535937000000     -0.483666000000      0.000030000000
H     4.061805000000     -2.563755000000     -0.000433000000
H     0.106129000000      1.316957000000     -0.000763000000
C     0.337581000000      0.258068000000     -0.000581000000
C    -0.653890000000     -0.642491000000     -0.000274000000
H    -0.427666000000     -1.702010000000     -0.000055000000
O    -4.309707000000      2.575056000000     -0.000039000000
H    -5.270004000000      2.671029000000      0.000250000000
O    -5.233473000000     -2.077428000000      0.000408000000
O     4.886222000000      1.894218000000      0.000494000000
H     5.797131000000      1.575092000000      0.002130000000
O     5.900900000000     -0.608818000000      0.000311000000
H     6.153530000000     -1.539617000000      0.003139000000


Products for ISO

A'4

C    -4.603775000000     -0.925744000000      0.032001000000
C    -3.261873000000     -1.283437000000      0.022910000000
C    -2.278969000000     -0.289259000000     -0.009056000000
C    -2.653486000000      1.060001000000     -0.035585000000
C    -3.999036000000      1.387865000000     -0.028666000000
C    -4.988560000000      0.408237000000      0.003153000000
H    -2.986859000000     -2.329242000000      0.043904000000
H    -1.926308000000      1.857706000000     -0.064872000000
H    -6.036921000000      0.683150000000      0.009118000000
C     1.566217000000     -0.327040000000     -0.009909000000
C     1.955546000000     -1.702047000000     -0.035934000000
C     2.563603000000      0.672420000000      0.011922000000
C     3.265724000000     -2.053431000000     -0.039113000000
H     1.201231000000     -2.474761000000     -0.054876000000
C     3.894488000000      0.337858000000      0.009012000000
H     2.251785000000      1.706545000000      0.031598000000
C     4.315662000000     -1.063855000000     -0.017399000000
H     3.573994000000     -3.090058000000     -0.059830000000
H     0.045137000000      1.169961000000      0.007727000000
C     0.193319000000      0.096907000000     -0.005966000000
C    -0.884207000000     -0.723087000000     -0.013680000000
H    -0.745269000000     -1.796777000000     -0.019961000000
O    -4.326294000000      2.719216000000     -0.056627000000
H    -5.284636000000      2.823743000000     -0.052151000000
O    -5.527206000000     -1.938176000000      0.070060000000
H    -6.419972000000     -1.574543000000      0.083819000000
O     4.912283000000      1.209291000000      0.029183000000
O     5.525503000000     -1.370412000000     -0.020205000000
C     4.591680000000      2.607262000000      0.063186000000
H     4.031559000000      2.884869000000     -0.828791000000
H     5.544111000000      3.124939000000      0.083127000000
H     4.019857000000      2.839222000000      0.960637000000


B'3

C     4.750471000000     -0.856507000000      0.049698000000
C     3.374888000000     -1.208645000000      0.040706000000
C     2.364502000000     -0.234151000000     -0.013057000000
C     2.743764000000      1.094869000000     -0.066178000000
C     4.139550000000      1.478344000000     -0.057160000000
C     5.142699000000      0.446248000000     -0.000403000000
H     3.116144000000     -2.258138000000      0.078358000000
H     2.025381000000      1.899796000000     -0.121192000000
H     6.186945000000      0.729493000000      0.005392000000
C    -1.506536000000     -0.326230000000     -0.004490000000
C    -1.902420000000     -1.663063000000     -0.049442000000
C    -2.493691000000      0.671228000000      0.028725000000
C    -3.251278000000     -1.992968000000     -0.059987000000
H    -1.171274000000     -2.458100000000     -0.077931000000
C    -3.840854000000      0.345200000000      0.016248000000
H    -2.185015000000      1.706182000000      0.063207000000
C    -4.219991000000     -1.002479000000     -0.029483000000
H    -3.566985000000     -3.028457000000     -0.095517000000
H     0.032052000000      1.181343000000      0.032937000000
C    -0.104799000000      0.105769000000      0.004622000000
C     0.969294000000     -0.693496000000     -0.016740000000
H     0.848276000000     -1.770267000000     -0.033989000000
O     4.456443000000      2.691258000000     -0.100028000000
O     5.616256000000     -1.904675000000      0.110734000000
H     6.527324000000     -1.586014000000      0.120431000000
O    -4.861411000000      1.250605000000      0.043714000000
O    -5.561450000000     -1.276438000000     -0.041999000000
H    -5.693604000000     -2.230840000000     -0.079745000000
C    -4.502066000000      2.633824000000      0.100885000000
H    -3.929678000000      2.843953000000      1.004133000000
H    -5.439302000000      3.180118000000      0.123552000000
H    -3.930171000000      2.917266000000     -0.782669000000


B'5

C     4.760282000000     -1.009130000000     -0.000075000000
C     3.352938000000     -1.348518000000      0.000203000000
C     2.376634000000     -0.376862000000      0.000217000000
C     2.779717000000      0.977291000000      0.000170000000
C     4.149579000000      1.332354000000     -0.000066000000
C     5.129635000000      0.383511000000     -0.000113000000
H     3.095488000000     -2.399254000000      0.000317000000
H     2.058865000000      1.781284000000      0.000412000000
H     6.178758000000      0.648421000000     -0.000219000000
C    -1.495130000000     -0.348849000000      0.000003000000
C    -1.921483000000     -1.676989000000      0.000317000000
C    -2.459260000000      0.671421000000     -0.000350000000
C    -3.277330000000     -1.976908000000      0.000271000000
H    -1.208734000000     -2.488889000000      0.000635000000
C    -3.813421000000      0.375076000000     -0.000444000000
H    -2.128388000000      1.700141000000     -0.000603000000
C    -4.223628000000     -0.964410000000     -0.000127000000
H    -3.616066000000     -3.005585000000      0.000557000000
H     0.079065000000      1.121063000000     -0.000139000000
C    -0.083177000000      0.049108000000      0.000125000000
C     0.967370000000     -0.781980000000      0.000281000000
H     0.811990000000     -1.854375000000      0.000257000000
O     4.404891000000      2.670366000000     -0.000027000000
H     5.357099000000      2.827201000000     -0.001240000000
O     5.630990000000     -1.911304000000     -0.000251000000
O    -4.813253000000      1.303673000000     -0.000877000000
O    -5.571107000000     -1.206491000000     -0.000136000000
H    -5.727812000000     -2.157923000000     -0.000729000000
C    -4.422152000000      2.679015000000      0.000822000000
H    -3.844779000000      2.911955000000      0.895333000000
H    -5.346666000000      3.247138000000      0.001267000000
H    -3.844441000000      2.914087000000     -0.892929000000


Cartesian coordinates of the RAF products in water:

Products for PIC with NO

C20

C     4.272771000000      1.223951000000      0.118831000000
C     2.925708000000      1.392870000000      0.376199000000
C     2.016760000000      0.356119000000      0.072113000000
C     2.501393000000     -0.845535000000     -0.484908000000
C     3.858572000000     -0.982058000000     -0.723291000000
C     4.758944000000      0.039852000000     -0.432652000000
H     2.571708000000      2.318901000000      0.807922000000
H     1.846290000000     -1.665618000000     -0.736153000000
H     5.817049000000     -0.084840000000     -0.630428000000
C    -1.819657000000      0.043551000000      0.018405000000
C    -2.154458000000      1.382354000000     -0.144238000000
C    -2.829124000000     -0.922708000000      0.000763000000
C    -3.485367000000      1.754884000000     -0.319868000000
H    -1.393831000000      2.148902000000     -0.151895000000
C    -4.150150000000     -0.553800000000     -0.168227000000
H    -2.592944000000     -1.974178000000      0.109673000000
C    -4.480663000000      0.794396000000     -0.327839000000
H    -3.756608000000      2.794393000000     -0.453625000000
H    -0.160255000000     -1.209528000000     -0.516514000000
C    -0.407865000000     -0.440477000000      0.211139000000
C     0.639637000000      0.582052000000      0.347218000000
H     0.382605000000      1.512731000000      0.832865000000
O     4.290069000000     -2.165848000000     -1.264397000000
H     5.246936000000     -2.142479000000     -1.379977000000
O     5.115986000000      2.261035000000      0.426141000000
H     6.023546000000      2.022457000000      0.206096000000
O    -5.125672000000     -1.516791000000     -0.184355000000
H    -5.983475000000     -1.096515000000     -0.325367000000
O    -5.811952000000      1.074357000000     -0.493301000000
H    -5.938163000000      2.022304000000     -0.616884000000
O     0.324432000000     -2.122838000000      1.575001000000
N    -0.326397000000     -1.119794000000      1.588529000000


C21

C    -4.129788000000     -0.827952000000     -0.912148000000
C    -2.817662000000     -1.156149000000     -0.588205000000
C    -2.044995000000     -0.238965000000      0.115789000000
C    -2.576002000000      0.986909000000      0.509138000000
C    -3.893300000000      1.283844000000      0.184384000000
C    -4.682069000000      0.387679000000     -0.529179000000
H    -2.412750000000     -2.111587000000     -0.892557000000
H    -1.990658000000      1.708875000000      1.061822000000
H    -5.706711000000      0.634322000000     -0.781408000000
C     1.760949000000      0.107974000000     -0.083524000000
C     2.303760000000     -1.192109000000      0.031912000000
C     2.648761000000      1.183301000000     -0.339629000000
C     3.667016000000     -1.399004000000     -0.095158000000
H     1.664193000000     -2.041782000000      0.213474000000
C     3.998515000000      0.968198000000     -0.467626000000
H     2.265175000000      2.190931000000     -0.431929000000
C     4.518393000000     -0.330369000000     -0.342160000000
H     4.085517000000     -2.393455000000     -0.007024000000
H     0.064425000000      1.422120000000     -0.035909000000
C     0.380650000000      0.391575000000      0.050580000000
C    -0.630242000000     -0.599688000000      0.463176000000
H    -0.396458000000     -1.612379000000      0.146452000000
O    -4.386849000000      2.495638000000      0.593352000000
H    -5.306510000000      2.585948000000      0.319670000000
O    -4.853807000000     -1.751665000000     -1.620931000000
H    -5.735396000000     -1.409633000000     -1.809125000000
O     4.839008000000      2.023071000000     -0.713339000000
H     5.747201000000      1.698846000000     -0.763073000000
O     5.870321000000     -0.446075000000     -0.480583000000
H     6.147408000000     -1.362734000000     -0.365430000000
O     0.264033000000     -1.415062000000      2.400221000000
N    -0.473001000000     -0.564026000000      1.990170000000


Products for ISO with NO

C'20

C    -4.377217000000     -0.787172000000     -1.110462000000
C    -3.043067000000     -1.115455000000     -0.961616000000
C    -2.195865000000     -0.284379000000     -0.194076000000
C    -2.735571000000      0.870001000000      0.413887000000
C    -4.075924000000      1.165849000000      0.245700000000
C    -4.916078000000      0.350581000000     -0.511195000000
H    -2.651100000000     -2.004568000000     -1.435780000000
H    -2.129930000000      1.534144000000      1.013282000000
H    -5.963829000000      0.597451000000     -0.632861000000
C     1.595486000000     -0.205432000000      0.389518000000
C     2.053760000000     -1.494285000000      0.626203000000
C     2.463987000000      0.762849000000     -0.119921000000
C     3.376223000000     -1.823785000000      0.344170000000
H     1.387431000000     -2.247089000000      1.028432000000
C     3.781407000000      0.436085000000     -0.407640000000
H     2.095613000000      1.763558000000     -0.292455000000
C     4.236427000000     -0.869503000000     -0.170017000000
H     3.750000000000     -2.824262000000      0.521860000000
H     0.029140000000      1.224148000000      0.556575000000
C     0.162207000000      0.150100000000      0.704430000000
C    -0.832485000000     -0.646153000000     -0.054153000000
H    -0.504503000000     -1.571767000000     -0.505452000000
O    -4.554363000000      2.297702000000      0.857350000000
H    -5.493884000000      2.401251000000      0.669804000000
O    -5.158529000000     -1.621641000000     -1.870645000000
H    -6.058785000000     -1.279954000000     -1.911663000000
O     4.702056000000      1.301813000000     -0.917243000000
O     5.546247000000     -1.133640000000     -0.469536000000
H     5.743083000000     -2.056431000000     -0.269513000000
C     4.258246000000      2.632932000000     -1.197202000000
H     3.458609000000      2.619024000000     -1.937735000000
H     5.123109000000      3.153071000000     -1.595274000000
H     3.918530000000      3.122510000000     -0.284893000000
O    -0.742454000000     -0.770000000000      2.598247000000
N     0.063379000000      0.017376000000      2.214934000000


C'21

C    -4.254441000000     -0.703338000000     -1.094519000000
C    -3.003299000000     -1.144955000000     -0.677602000000
C    -2.207716000000     -0.304974000000      0.095450000000
C    -2.661766000000      0.960368000000      0.461785000000
C    -3.919498000000      1.372416000000      0.042549000000
C    -4.726636000000      0.553086000000     -0.739233000000
H    -2.659356000000     -2.128855000000     -0.965904000000
H    -2.065991000000      1.622958000000      1.073965000000
H    -5.703511000000      0.890103000000     -1.065459000000
C     1.599457000000     -0.234495000000      0.135423000000
C     2.033915000000     -1.577416000000      0.122951000000
C     2.570526000000      0.786468000000     -0.062655000000
C     3.370366000000     -1.880305000000     -0.077418000000
H     1.335125000000     -2.385841000000      0.277364000000
C     3.894855000000      0.477093000000     -0.272235000000
H     2.242138000000      1.815547000000     -0.051309000000
C     4.302645000000     -0.873371000000     -0.279392000000
H     3.706915000000     -2.909429000000     -0.083666000000
H     0.026766000000      1.201494000000      0.388689000000
C     0.253274000000      0.146198000000      0.329750000000
C    -0.865001000000     -0.802541000000      0.544566000000
H    -0.656812000000     -1.771673000000      0.095103000000
O    -4.339394000000      2.620198000000      0.427755000000
H    -5.227028000000      2.784466000000      0.089622000000
O    -4.999766000000     -1.555711000000     -1.868401000000
H    -5.836050000000     -1.139139000000     -2.105541000000
O     4.886714000000      1.388532000000     -0.481972000000
O     5.626868000000     -1.120647000000     -0.491714000000
H     5.780916000000     -2.072933000000     -0.498030000000
C     4.498432000000      2.763884000000     -0.549327000000
H     3.783683000000      2.917919000000     -1.357584000000
H     5.409781000000      3.317050000000     -0.750945000000
H     4.071185000000      3.087796000000      0.399414000000
O    -1.686702000000     -0.688553000000      2.704876000000
N    -0.774316000000     -1.086962000000      2.049912000000


Products for PIC with NO2

C20

C     4.231419000000     -1.236780000000     -0.552191000000
C     2.928806000000     -1.111240000000     -0.994042000000
C     2.014232000000     -0.302739000000     -0.279871000000
C     2.458717000000      0.369944000000      0.880583000000
C     3.771592000000      0.223890000000      1.291145000000
C     4.675495000000     -0.574461000000      0.591335000000
H     2.610261000000     -1.635967000000     -1.884227000000
H     1.806013000000      1.002614000000      1.463112000000
H     5.699167000000     -0.677519000000      0.929610000000
C    -1.737131000000     -0.098331000000     -0.074466000000
C    -1.866972000000     -1.469237000000     -0.251604000000
C    -2.860127000000      0.663364000000      0.258976000000
C    -3.110783000000     -2.080635000000     -0.103769000000
H    -1.008462000000     -2.078148000000     -0.497323000000
C    -4.093046000000      0.058307000000      0.401875000000
H    -2.783626000000      1.732479000000      0.409608000000
C    -4.220811000000     -1.321934000000      0.216444000000
H    -3.223256000000     -3.148532000000     -0.241418000000
H    -0.128019000000      1.008386000000      0.792760000000
C    -0.399242000000      0.596558000000     -0.175808000000
C     0.687318000000     -0.220421000000     -0.768439000000
H     0.454610000000     -0.744396000000     -1.685720000000
O     4.154645000000      0.899239000000      2.422977000000
H     5.083047000000      0.726478000000      2.616220000000
O     5.075108000000     -2.038346000000     -1.279151000000
H     5.947184000000     -2.053284000000     -0.869933000000
O    -5.187646000000      0.816173000000      0.727531000000
H    -5.956218000000      0.237344000000      0.808811000000
O    -5.481844000000     -1.833929000000      0.377656000000
H    -5.475251000000     -2.788023000000      0.237509000000
N    -0.031819000000      2.885038000000     -0.558827000000
O    -0.611127000000      1.767614000000     -1.057766000000
O    -0.159076000000      3.794949000000     -1.292276000000


C21

C    -3.895312000000     -0.741300000000      1.329055000000
C    -2.608774000000     -0.211274000000      1.358232000000
C    -1.947887000000      0.010781000000      0.157266000000
C    -2.553075000000     -0.277629000000     -1.062772000000
C    -3.840366000000     -0.797045000000     -1.063495000000
C    -4.521683000000     -1.037936000000      0.126141000000
H    -2.141162000000      0.019390000000      2.305691000000
H    -2.043032000000     -0.100264000000     -1.999907000000
H    -5.526710000000     -1.443075000000      0.113524000000
C     1.849592000000     -0.305623000000     -0.040472000000
C     2.402174000000      0.802597000000      0.644073000000
C     2.735463000000     -1.307071000000     -0.512943000000
C     3.767663000000      0.898860000000      0.842741000000
H     1.764129000000      1.591890000000      1.014034000000
C     4.090192000000     -1.202805000000     -0.309765000000
H     2.346436000000     -2.168320000000     -1.040017000000
C     4.617170000000     -0.093118000000      0.368585000000
H     4.193276000000      1.747470000000      1.362911000000
H     0.111037000000     -1.299015000000     -0.836639000000
C     0.464165000000     -0.445488000000     -0.275946000000
C    -0.540501000000      0.545841000000      0.184765000000
H    -0.306159000000      0.923482000000      1.179174000000
O    -4.415255000000     -1.065473000000     -2.278518000000
H    -5.306748000000     -1.410747000000     -2.153684000000
O    -4.517936000000     -0.953319000000      2.531224000000
H    -5.398281000000     -1.319577000000      2.387420000000
O     4.927998000000     -2.184455000000     -0.774173000000
H     5.837330000000     -1.959603000000     -0.539989000000
O     5.974988000000     -0.069472000000      0.516516000000
H     6.254146000000      0.741644000000      0.957803000000
N    -0.829262000000      2.849983000000     -0.103176000000
O    -0.440794000000      1.712289000000     -0.726817000000
O    -0.790543000000      3.772193000000     -0.830843000000


Products for ISO with NO2

C'20

C     4.449007000000     -1.517669000000      0.146899000000
C     3.115170000000     -1.650263000000     -0.188884000000
C     2.241840000000     -0.550411000000     -0.040825000000
C     2.749683000000      0.672379000000      0.451179000000
C     4.088454000000      0.765297000000      0.785693000000
C     4.955826000000     -0.316506000000      0.639495000000
H     2.745558000000     -2.593862000000     -0.566345000000
H     2.120586000000      1.541907000000      0.574020000000
H     6.002873000000     -0.225503000000      0.902524000000
C    -1.550122000000     -0.129111000000     -0.353821000000
C    -2.003805000000     -0.896291000000     -1.418684000000
C    -2.417484000000      0.202036000000      0.689206000000
C    -3.322180000000     -1.338187000000     -1.441189000000
H    -1.339494000000     -1.149242000000     -2.234450000000
C    -3.730888000000     -0.247537000000      0.675140000000
H    -2.054347000000      0.807617000000      1.506983000000
C    -4.181897000000     -1.021957000000     -0.402115000000
H    -3.693699000000     -1.934692000000     -2.265038000000
H     0.022948000000      0.982555000000      0.570665000000
C    -0.131206000000      0.363993000000     -0.312514000000
C     0.880990000000     -0.719158000000     -0.400156000000
H     0.564683000000     -1.679460000000     -0.780361000000
O     4.541120000000      1.967386000000      1.268179000000
H     5.478617000000      1.901635000000      1.483800000000
O     5.260680000000     -2.610773000000     -0.023277000000
H     6.164140000000     -2.389970000000      0.228772000000
O    -4.650561000000      0.011298000000      1.647836000000
O    -5.487704000000     -1.433589000000     -0.368724000000
H    -5.677004000000     -1.956989000000     -1.156099000000
C    -4.216998000000      0.802068000000      2.758822000000
H    -3.398665000000      0.308847000000      3.283118000000
H    -5.078105000000      0.884653000000      3.413861000000
H    -3.908409000000      1.792737000000      2.425446000000
N    -0.196752000000      2.539303000000     -1.187980000000
O     0.112879000000      1.254688000000     -1.478163000000
O    -0.042757000000      3.238808000000     -2.120913000000


C'21

C    -4.601969000000     -0.361687000000     -0.167684000000
C    -3.342424000000     -0.947091000000     -0.148152000000
C    -2.217377000000     -0.138188000000     -0.282817000000
C    -2.352336000000      1.235913000000     -0.448053000000
C    -3.625991000000      1.794767000000     -0.465129000000
C    -4.761841000000      1.010202000000     -0.320397000000
H    -3.262939000000     -2.018433000000     -0.032811000000
H    -1.496033000000      1.882449000000     -0.573123000000
H    -5.749136000000      1.455882000000     -0.332987000000
C     1.631206000000     -0.166815000000     -0.170983000000
C     2.049508000000     -1.272626000000     -0.944044000000
C     2.627736000000      0.716657000000      0.329744000000
C     3.393391000000     -1.474950000000     -1.209781000000
H     1.328010000000     -1.977004000000     -1.331681000000
C     3.961298000000      0.509444000000      0.059513000000
H     2.312993000000      1.562447000000      0.923646000000
C     4.351791000000     -0.600081000000     -0.718915000000
H     3.717387000000     -2.321552000000     -1.802471000000
H     0.045020000000      0.934924000000      0.769376000000
C     0.277801000000      0.096631000000      0.130353000000
C    -0.836463000000     -0.757969000000     -0.352859000000
H    -0.657044000000     -1.072722000000     -1.380494000000
O    -3.711963000000      3.153378000000     -0.626358000000
H    -4.635787000000      3.428744000000     -0.613612000000
O    -5.683980000000     -1.192836000000     -0.032459000000
H    -6.500439000000     -0.684240000000     -0.097970000000
O     4.979541000000      1.307856000000      0.489163000000
O     5.688051000000     -0.754684000000     -0.951231000000
H     5.834220000000     -1.539933000000     -1.492062000000
C     4.617698000000      2.467027000000      1.245195000000
H     3.976471000000      3.120721000000      0.653934000000
H     5.550077000000      2.972351000000      1.474290000000
H     4.115688000000      2.180535000000      2.168985000000
N    -0.930495000000     -2.150834000000      1.646382000000
O    -0.826509000000     -2.094512000000      0.305882000000
O    -1.082292000000     -1.111169000000      2.180798000000


Cartesian coordinates of the SET products in water:

NO-

O     0.000000000000      0.000000000000      0.578251000000
N     0.000000000000      0.000000000000     -0.660858000000

NO2-

N     0.000000000000      0.000000000000      0.460441000000
O     0.000000000000      1.051579000000     -0.201443000000
O     0.000000000000     -1.051579000000     -0.201443000000


Product from PIC

C     4.285324000000     -1.059309000000     -0.024503000000
C     2.922367000000     -1.313588000000     -0.026991000000
C     2.019165000000     -0.241160000000     -0.003363000000
C     2.490809000000      1.080698000000      0.026824000000
C     3.855878000000      1.304346000000      0.035169000000
C     4.763969000000      0.246008000000      0.009189000000
H     2.565645000000     -2.333918000000     -0.050919000000
H     1.824925000000      1.930217000000      0.048301000000
H     5.830060000000      0.440696000000      0.013486000000
C    -1.791777000000      0.010023000000     -0.017872000000
C    -2.271688000000     -1.337433000000      0.032064000000
C    -2.725690000000      1.071876000000     -0.054963000000
C    -3.610669000000     -1.596624000000      0.054983000000
H    -1.581305000000     -2.165738000000      0.057747000000
C    -4.068241000000      0.818819000000     -0.039008000000
H    -2.379606000000      2.095507000000     -0.095982000000
C    -4.525540000000     -0.531417000000      0.025668000000
H    -3.993864000000     -2.606931000000      0.099789000000
H    -0.186708000000      1.397212000000     -0.040750000000
C    -0.413714000000      0.339263000000     -0.022190000000
C     0.613715000000     -0.570600000000     -0.007852000000
H     0.394269000000     -1.629540000000     -0.003118000000
O     4.284334000000      2.602560000000      0.070005000000
H     5.247897000000      2.637623000000      0.076956000000
O     5.130121000000     -2.133303000000     -0.055128000000
H     6.048097000000     -1.838229000000     -0.058836000000
O    -4.959863000000      1.828333000000     -0.075587000000
H    -5.863080000000      1.483285000000     -0.057603000000
O    -5.838360000000     -0.687227000000      0.056978000000
H    -6.099629000000     -1.618507000000      0.117869000000


Product from ISO

C    -4.628342000000     -0.944765000000      0.034393000000
C    -3.285504000000     -1.292058000000      0.029207000000
C    -2.311494000000     -0.285083000000     -0.004899000000
C    -2.691702000000      1.065117000000     -0.038468000000
C    -4.038293000000      1.381845000000     -0.037330000000
C    -5.016693000000      0.389455000000     -0.002870000000
H    -2.998172000000     -2.334143000000      0.055711000000
H    -1.969814000000      1.867020000000     -0.070151000000
H    -6.067201000000      0.656921000000     -0.000703000000
C     1.510694000000     -0.290053000000     -0.000411000000
C     1.898021000000     -1.666633000000     -0.032737000000
C     2.510311000000      0.711520000000      0.023768000000
C     3.216328000000     -2.014343000000     -0.044540000000
H     1.151868000000     -2.445582000000     -0.050948000000
C     3.838073000000      0.372343000000      0.014415000000
H     2.202875000000      1.746082000000      0.048826000000
C     4.202373000000     -1.013971000000     -0.023577000000
H     3.531106000000     -3.049019000000     -0.072268000000
H    -0.002900000000      1.200424000000      0.019054000000
C     0.154755000000      0.129603000000      0.003984000000
C    -0.927990000000     -0.708043000000     -0.005301000000
H    -0.777835000000     -1.779355000000     -0.009370000000
O    -4.378065000000      2.706540000000     -0.073129000000
H    -5.337248000000      2.805059000000     -0.071548000000
O    -5.545025000000     -1.958803000000      0.076170000000
H    -6.441020000000     -1.602490000000      0.088177000000
O     4.869310000000      1.221382000000      0.034031000000
O     5.493659000000     -1.281497000000     -0.039672000000
H     5.658399000000     -2.235500000000     -0.079375000000
C     4.567920000000      2.626763000000      0.071395000000
H     4.009016000000      2.909697000000     -0.819334000000
H     5.528612000000      3.127906000000      0.088835000000
H     4.002539000000      2.861831000000      0.971731000000


Cartesian coordinates of the SPLET products in water:

Product for PIC

C    -4.299090000000     -1.028442000000      0.000175000000
C    -2.937716000000     -1.304601000000      0.000099000000
C    -2.010272000000     -0.257591000000     -0.000057000000
C    -2.473199000000      1.064455000000     -0.000138000000
C    -3.836545000000      1.311337000000     -0.000072000000
C    -4.770733000000      0.278083000000      0.000079000000
H    -2.604968000000     -2.333970000000      0.000181000000
H    -1.796977000000      1.906862000000     -0.000250000000
H    -5.833469000000      0.488858000000      0.000135000000
C     1.860996000000     -0.051437000000     -0.000067000000
C     2.369556000000     -1.355323000000     -0.000125000000
C     2.782994000000      1.014136000000     -0.000168000000
C     3.737622000000     -1.597429000000      0.000034000000
H     1.692814000000     -2.199917000000     -0.000321000000
C     4.135092000000      0.767122000000      0.000065000000
H     2.429605000000      2.038705000000     -0.000279000000
C     4.677473000000     -0.546923000000      0.000544000000
H     4.109546000000     -2.614763000000     -0.000236000000
H     0.217784000000      1.330070000000     -0.000147000000
C     0.435310000000      0.266845000000     -0.000117000000
C    -0.584851000000     -0.606861000000     -0.000090000000
H    -0.381098000000     -1.671667000000     -0.000052000000
O    -4.238184000000      2.625478000000     -0.000160000000
H    -5.200529000000      2.675731000000     -0.000046000000
O    -5.158993000000     -2.099408000000      0.000347000000
H    -6.071354000000     -1.789199000000      0.000483000000
O     5.048961000000      1.803635000000      0.000167000000
H     5.912532000000      1.357457000000      0.000684000000
O     5.974003000000     -0.693253000000     -0.000494000000


Product for ISO

C    -4.637604000000     -0.915761000000      0.057454000000
C    -3.299615000000     -1.287951000000      0.046169000000
C    -2.300438000000     -0.310516000000     -0.012121000000
C    -2.669239000000      1.040357000000     -0.064192000000
C    -4.011476000000      1.382740000000     -0.053105000000
C    -5.015182000000      0.419405000000      0.004377000000
H    -3.040036000000     -2.337361000000      0.087265000000
H    -1.935858000000      1.831628000000     -0.118215000000
H    -6.060219000000      0.705491000000      0.011810000000
C     1.575852000000     -0.372294000000     -0.019242000000
C     2.000789000000     -1.702653000000     -0.065839000000
C     2.565854000000      0.631614000000      0.019542000000
C     3.350899000000     -2.018646000000     -0.070781000000
H     1.276582000000     -2.506366000000     -0.100312000000
C     3.909770000000      0.318013000000      0.013472000000
H     2.245290000000      1.664153000000      0.054651000000
C     4.367453000000     -1.036801000000     -0.031853000000
H     3.666312000000     -3.054617000000     -0.107638000000
H     0.029638000000      1.115899000000      0.005526000000
C     0.175973000000      0.040409000000     -0.011145000000
C    -0.903442000000     -0.759775000000     -0.018004000000
H    -0.774782000000     -1.836404000000     -0.022225000000
O    -4.323728000000      2.720168000000     -0.106482000000
H    -5.280947000000      2.832834000000     -0.098250000000
O    -5.572238000000     -1.920381000000      0.124077000000
H    -6.459638000000     -1.544428000000      0.139109000000
O     4.916427000000      1.260149000000      0.048523000000
O     5.632685000000     -1.326575000000     -0.036385000000
C     4.521345000000      2.627154000000      0.120749000000
H     3.944634000000      2.912127000000     -0.760441000000
H     5.441217000000      3.203350000000      0.155525000000
H     3.936999000000      2.815035000000      1.022440000000
